# Spatiotemporal genomic profiling of intestinal metaplasia reveals clonal dynamics of gastric cancer progression

**DOI:** 10.1016/j.ccell.2023.10.004

**Published:** 2023-12-11

**Authors:** Kie Kyon Huang, Haoran Ma, Roxanne Hui Heng Chong, Tomoyuki Uchihara, Benedict Shi Xiang Lian, Feng Zhu, Taotao Sheng, Supriya Srivastava, Su Ting Tay, Raghav Sundar, Angie Lay Keng Tan, Xuewen Ong, Minghui Lee, Shamaine Wei Ting Ho, Tom Lesluyes, Hassan Ashktorab, Duane Smoot, Peter Van Loo, Joy Shijia Chua, Kalpana Ramnarayanan, Louis Ho Shing Lau, Takuji Gotoda, Hyun Soo Kim, Tiing Leong Ang, Christopher Khor, Jonathan Wei Jie Lee, Stephen Kin Kwok Tsao, Wei Lyn Yang, Ming Teh, Hyunsoo Chung, Jimmy Bok Yan So, Khay Guan Yeoh, Patrick Tan, Singapore Gastric Cancer Consortium

**Affiliations:** 1 Program in Cancer and Stem Cell Biology, Duke-NUS Medical School, Singapore 169857, Singapore; 2 Department of Medicine, Yong Loo Lin School of Medicine, National University of Singapore, Singapore 119228, Singapore; 3 Genome Institute of Singapore, Agency for Science, Technology and Research, Singapore, Singapore; 4 Department of Haematology-Oncology, National University Health System, Singapore 119074, Singapore; 5 The Francis Crick Institute, London, UK; 6 Department of Medicine, Howard University, Washington, DC, USA; 7 Department of Internal Medicine, Meharry Medical College, Nashville, TN, USA; 8 Department of Genetics, The University of Texas MD Anderson Cancer Center, Houston, TX, USA; 9 Department of Genomic Medicine, The University of Texas MD Anderson Cancer Center, Houston, TX, USA; 10 Department of Medicine and Therapeutics, Faculty of Medicine, The Chinese University of Hong Kong, Hong Kong, Hong Kong; 11 Division of Gastroenterology and Hepatology, Department of Medicine, Nihon University School of Medicine, Tokyo, Japan; 12 Department of Internal Medicine, Yonsei University Wonju College of Medicine, Seoul, Korea; 13 Department of Gastroenterology & Hepatology, Changi General Hospital, Singapore 529889, Singapore; 14 Department of Gastroenterology & Hepatology, Singapore General Hospital, Singapore 169854, Singapore; 15 iHealthtech, National University of Singapore, Singapore, Singapore; 16 SynCTI, National University of Singapore, Singapore 117599, Singapore; 17 Department of Gastroenterology & Hepatology, Tan Tock Seng Hospital, Singapore 308433, Singapore; 18 Department of Pathology, Yong Loo Lin School of Medicine, National University of Singapore, Singapore 119228, Singapore; 19 Department of Internal Medicine, Seoul National University Hospital, Seoul, Korea; 20 Department of Surgery, Yong Loo Lin School of Medicine, National University of Singapore, Singapore 119228, Singapore; 21 NUS Centre for Cancer Research, Yong Loo Lin School of Medicine, National University of Singapore, Singapore, Singapore; 22 Division of Surgical Oncology, National University Cancer Institute of Singapore (NCIS), Singapore, Singapore; 23 Department of Gastroenterology & Hepatology, National University Hospital, Singapore 119074, Singapore; 24 Cancer Science Institute of Singapore, National University of Singapore, Singapore 117599, Singapore; 25 Department of Physiology, Yong Loo Lin School of Medicine, National University of Singapore, Singapore 117593, Singapore; 26 Cellular and Molecular Research, National Cancer Centre, Singapore, Singapore; 27 Singhealth/Duke-NUS Institute of Precision Medicine, National Heart Centre Singapore, Singapore 168752, Singapore

**Keywords:** gastric cancer, intestinal metaplasia, pre-cancer, targeted DNA sequencing, transcriptome sequencing, single-cell sequencing, spatial transcriptomics, cancer screening, cell-of-origin

## Abstract

Intestinal metaplasia (IM) is a pre-malignant condition of the gastric mucosa associated with increased gastric cancer (GC) risk. Analyzing 1,256 gastric samples (1,152 IMs) across 692 subjects from a prospective 10-year study, we identify 26 IM driver genes in diverse pathways including chromatin regulation (*ARID1A*) and intestinal homeostasis (*SOX9*). Single-cell and spatial profiles highlight changes in tissue ecology and IM lineage heterogeneity, including an intestinal stem-cell dominant cellular compartment linked to early malignancy. Expanded transcriptome profiling reveals expression-based molecular subtypes of IM associated with incomplete histology, antral/intestinal cell types, *ARID1A* mutations, inflammation, and microbial communities normally associated with the healthy oral tract. We demonstrate that combined clinical-genomic models outperform clinical-only models in predicting IMs likely to transform to GC. By highlighting strategies for accurately identifying IM patients at high GC risk and a role for microbial dysbiosis in IM progression, our results raise opportunities for GC precision prevention and interception.

## Highlights

• Genomic profiling reveals 26 intestinal metaplasia (IM) driver genes including *SOX9*
• IM lineage heterogeneity harbors a stem-cell subpopulation linked to malignancy
• IM molecular subtypes are associated with inflammation and microbial communities
• Genomic-clinical models improve identification of IMs at risk of GC progression

## Graphical abstract

**Figure:**
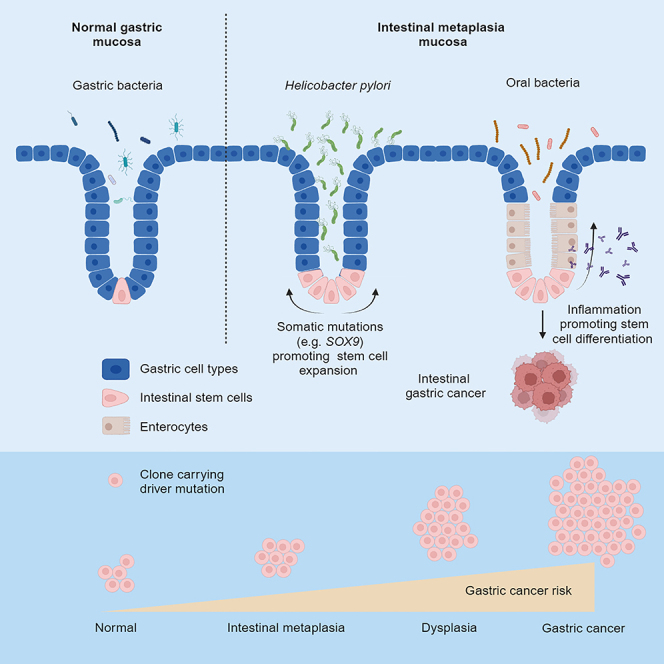

## Teaser

Intestinal metaplasia (IM) is a pre-malignant condition linked to gastric cancer (GC). Huang et al. analyze a large prospective IM cohort, identifying driver genes, stem cell populations linked to GC, and molecular subtypes associated with inflammation and oral microbes. These results facilitate the use of genomics in GC precision prevention.

## Introduction

Gastric cancer (GC) is a major cause of global cancer burden.^1^ Despite an overall decline in age-adjusted incidence, GC still ranks fifth in incidence and fourth in mortality.^2^ In countries with high GC prevalence such as Japan and South Korea, population screening has resulted in improved outcomes due to early detection.^3^ However, in many countries such as Singapore where GC incidence is moderate, population screening is not cost-effective.^4^ There is thus a need to better understand the pathogenesis of GC to guide precision prevention efforts.

The stomach is a complex organ with distinct anatomical regions (antrum, body, and cardia) harboring different cell types and functionalities.^5^ An important step in GC carcinogenesis is intestinal metaplasia (IM),^6^ a pre-malignant condition where cells lining the stomach are replaced by cells with characteristics similar to the small intestine. IM patients have increased GC risk (6-fold^7^). However, it remains unclear if IM cells represent direct precursors of malignancy, or if the presence of IM reflects bystander tissue damage caused by *Helicobacter pylori* (Hp) and chronic inflammation.^8^^,^^9^^,^^10^ Some groups have proposed that IM cells, being post-mitotic and differentiated, are unlikely to cause cancer^8^^,^^10^ and that GC may emerge from other gastric stem cell populations.^8^^,^^10^ IMs are also heterogeneous between and within patients. For example, IMs can exhibit either Type I “complete” histology (small intestinal-type mucosa and mature absorptive cells, goblet cells and brush borders) or Type III “incomplete” histology (colonic epithelium and columnar intermediate cells in various stages of differentiation). Besides IM, other variants of stomach metaplasia have also been reported such as spasmolytic polypeptide-expressing metaplasia (SPEM).^9^

To date, only a handful of studies have examined genomic and molecular features of IM.^11^^,^^12^^,^^13^ Here, we performed a comprehensive analysis of IMs sampled from a prospective clinical study, leveraging high-depth targeted DNA sequencing, transcriptome sequencing, and recently developed single-cell and spatial transcriptomic platforms.

## Results

### Driver gene landscape of gastric premalignancy

**Figure 1:**
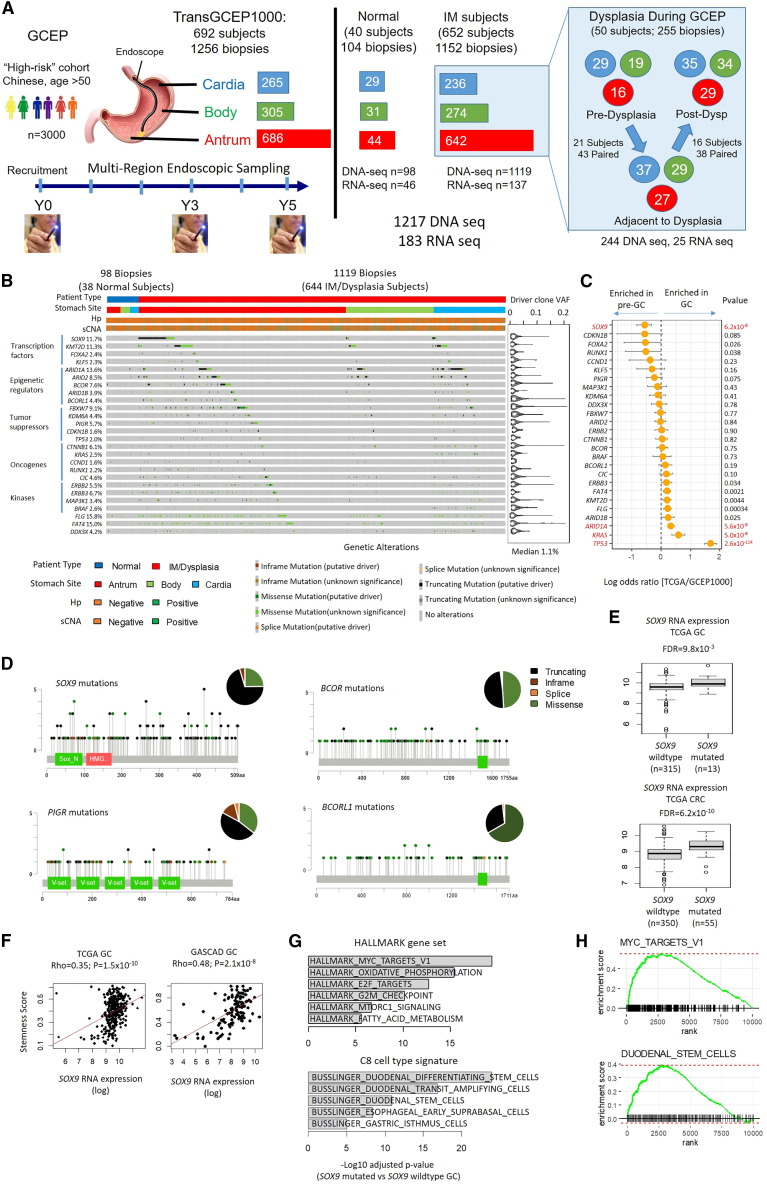
Genomic profiles of gastric pre-malignancy (A) GCEP1000 translational study overview. 1,256 gastric biopsies from multiple stomach sites were analyzed from 692 GCEP subjects. (Right) Samples which were longitudinally matched from the same subjects, from either pre-dysplasia to dysplasia (adjacent) or dysplasia (adjacent) to post-dysplasia. (B) Predicted IM driver genes. (Right) Violin plots indicate median VAFs of somatic mutations. (C) Log ORs of driver gene mutation frequencies in TCGA (GC) vs. GCEP1000 (pre-malignancy). Left shifted genes are mutated more frequently in pre-malignancy, while right-shifted genes are mutated more frequently in GC. p values utilized Fisher’s exact tests. (D) Distributions and categories of protein altering mutations in *SOX9*, *PIGR*, *BCOR*, and *BCORL1*. Pie charts indicate percentages of different types of mutations. (E) Boxplot comparing *SOX9* expression levels in *SOX9*-mutated and *SOX9*-wildtype GC (upper) and CRC (lower). FDR values utilized DESeq2. Box, median +/− interquartile range (IQR). Whiskers, 1.5× IQR. (F) Correlation between *SOX9* expression and stemness scores in TCGA GCs (left) and GASCAD cohort (right). p values utilized Spearman’s correlation coefficient. (G) GSEA of *SOX9*-mutated vs. *SOX9*-wildtype GCs using HALLMARK (upper) and Busslinger et al. datasets^26^ (lower). Adjusted p values utilized fgsea. (H) Enrichment of MYC target V1 pathway genes and duodenal stem cell signatures in *SOX9* mutated GCs. See also Figures S1–S3, Tables S1, S2, and S3.

The Gastric Cancer Epidemiology Program (GCEP) is a prospective multi-center longitudinal cohort study, monitoring 2,980 Chinese participants aged ≥50 from 2004 to 2015.^14^ At enrollment, GCEP subjects underwent screening gastroscopies with standardized gastric mucosal sampling at multiple stomach regions (antrum, body, cardia) and surveillance endoscopies at years 3 and 5. For this study (“TransGCEP1000”), we performed high-depth (>1000×) targeted DNA sequencing of 277 cancer genes on 1,217 frozen endoscopic biopsies from 682 unique subjects (1,119 samples from 644 subjects with IM; 98 samples from 38 control subjects without IM) (Figure 1A and Table S1). The 277-gene panel was designed specifically for GC by curating for genes mutated in gastric, esophageal, and colorectal cancers reported by TCGA and other studies,^15^^,^^16^^,^^17^ augmented with additional driver genes mutated in normal or inflamed tissues^18^^,^^19^^,^^20^ (Table S2). Average coverage was 1046× to confidently identify small clonal events, a considerably deeper sequencing depth compared to our previous study where only one IM driver gene (*FBXW7*) was identified at an average depth of 365×.^11^We identified 23,575 somatic mutations across the 1,217 samples with a median variant allele frequency (VAF) of 1.0% (range 0.075%–36.7%; compared to paired blood samples). Reinforcing the importance of high-sequencing depth to detect small clones, downsampling simulations revealed that 83% of driver mutations would have been missed if we had used a coverage of 216× (a typical depth for whole-exome sequencing; WES). The mutation calls were validated using two other mutation callers (STAR methods).

IM mutation rates were significantly higher compared to normal gastric samples (9.6 vs. 1.8 mutations/Mb; Wilcoxon test p < 2.2 × 10^−16^) (Figure S1A) and correlated weakly with subject age (r = 0.26, Pearson’s correlation test, p value < 2.2 × 10^−16^) (Figure S1B). Most IM samples exhibited single base substitution (SBS) mutational signatures SBS1 (aging; 97% of IM biopsies), SBS18 (oxidative stress; 3.2%), SBS5 (clock-like signature; 2.6%), SBS3+8 (homologous recombination; 1.1%), and SBS17 (unknown etiology; 1.1%) (Figures S1C–S1H). Besides coding exons, our panel included Hp genes and ∼5000 single-nucleotide polymorphisms (SNPs) distributed across the genome, allowing us to infer Hp infection status and somatic copy number alterations (sCNAs). High Hp burden (>10X coverage) was observed in 6.1% of biopsies from IM subjects (68/1,119) compared to 1.0% of normal samples (1/98; Fisher’s test p value 0.037) (Figure S2A). Four significant sCNA regions were identified (7q36 and 8q24 amplifications, 8p23 and 11p15 deletions) (Figure S2B). The common amplified 8q region (chr8:125300000-133800000) included the *MYC* oncogene,^11^ while the 7q region (chr7:141800000-144000000) contained genes such as *PRSS1* that may promote GC growth^21^ (Figure S2C).

Using dNdScv22 to identify genes under positive selection, we identified 26 candidate driver genes (q < 0.15) (Figure 1B). Of these, 22 (85%) were also identified by two other driver gene algorithms (STAR methods and Table S2). These included credentialed oncogenes (e.g., *KRAS*, *ERBB2*, *ERBB3*, and *BRAF*) and tumor suppressors (e.g., *ARID1A* and *TP53*) including *FBXW7* which we previously reported.^11^ Of the 26 driver genes, 22 were enriched in IMs relative to normal (Odds ratio (OR) > 2), including *SOX9* (OR 14.0, Fisher’s test p value 9.9 × 10-5), *ARID1A* (OR 2.5, Fisher’s test p value 0.021), and *FBXW7* (OR 10.6, Fisher’s test p value 1.4 × 10^−3^) (Table S3). While most of the IM driver genes were significantly enriched in antral IMs, *ARID1A* mutations occurred more commonly in body/cardia IMs possibly suggesting distinct selective pressures at different stomach sites (Table S3). Many of the driver events were present at relatively low VAFs (median 1.1%, range 0.093%–21.0%). Notably, *TP53* was mutated in 2.0% (24/1217) of premalignant samples compared to 48.9% of GCs (TCGA 213/436; Fisher’s test, OR 0.02, p value < 2.2 × 10^−16^) (Figure 1C), suggesting that *TP53* mutations are likely to occur later in gastric tumorigenesis after IM onset.

IM driver genes included *SOX9*, *PIGR*, *BCOR*, *BCORL1*, and *KLF5* (Figures 1D and S2D). In an independent validation cohort of 150 IMs from Singapore and other Asian countries (33 SG, 16 Hong Kong, 85 Korean, and 16 Japanese), we also observed *SOX9* (4/33 SG; 10/117 non-SG), *ARID1A* (5/33, 18/117), *ARID2* (4/33; 15/117), and *FBXW7* (2/33; 20/117) mutations (Table S3). *SOX9* encodes a transcription factor controlling intestinal crypt homeostasis by blocking intestinal differentiation and promoting an intestinal stem cell-like program, and *SOX9* mutations have been reported in 29% of genome stable colorectal cancers (CRC).^16^^,^^23^ We noted a higher prevalence of *SOX9* mutations in GCEP compared to TCGA GCs (Fisher’s test p value 6.2 × 10^−8^) (Figure 1C). Similarly, low *SOX9* mutation rates were observed in Asian GC patients including TCGA GC (Asian) samples (2.2%, 2/89) and GC cohorts from Hong Kong^17^ (2%, 2/100) and Singapore^24^ (3.3%, 7/213) (Table S3). In GCEP, similar to CRC the majority of *SOX9* mutations were C-terminal truncating exon 3 mutations (truncating mutations: 119/173; 69%; truncating mutations at exon 3; 80/119; 67.2%) (Figure 1D) and more common in antral biopsies compared to body/cardia (Figure S3A; 18.4% vs. 5.1%; Fisher’s test p value 6.9 × 10^−12^).

Mining TCGA data, we found that *SOX9* C-terminal truncating mutations were significantly associated with higher *SOX9* RNA expression in both GC and CRC (GC: log2FoldChange 0.86; adjusted p value 9.8 × 10^−3^; CRC: log2FoldChange 0.73; adjusted p value 6.2 × 10^−10^) (Figure 1E), supporting previous reports that *SOX9* truncating exon 3 mutations are associated with higher SOX9 protein expression.^25^ High *SOX9* expression was observed in chromosomally unstable (CIN) GCs, a molecular subtype associated with IM (Wilcoxon test p value 1.6 × 10^−7^ for TCGA and 4.9 × 10^−6^ for GASCAD) (Figure S3B). Increased *SOX9* RNA expression was significantly correlated with stemness scores in GC (TCGA GC: Spearman rho 0.35, p value 1.5 × 10^−10^, GASCAD: Spearman rho 0.48, p value 2.1 × 10^−8^), with *SOX9* mutated GCs showing expression signatures of oxidative phosphorylation (Normalized enrichment score, NES 2.6; adjusted p value 3.6 × 10^−16^) and MYC pathway targets (NES 2.8; adjusted p value 3.2 × 10^−20^) (Figures 1F–1H).

Using CRISPR/Cas9 genome editing, we deleted the *SOX9* C-terminus (Exon 3) in two human gastric non-malignant epithelial cell lines (GES-1, HFE-145) and one GC cell line (SNU-484) preserving the 3′ untranslated region (UTR) (Figure S3C). Consistent with observations in primary samples, loss of the *SOX9* C-terminus resulted in higher *SOX9* gene expression *in vitro* (Figure S3D). Inspection of RNA-seq (RNA-sequencing) reads confirmed that *SOX9* expression was largely driven by the truncated *SOX9* allele in the C-terminus deleted clones (Figure S3E). While *SOX9* C-terminal loss did not affect cell proliferation (Figure S3F), we observed increased expression of gastric stem cell markers such as *LGR5* (log2FoldChange 2.9, adjusted p value 2.4 × 10^−5^) and *AQP5* (log2FoldChange 4.9, adjusted p value 0.038), with a corresponding increase in stemness scores in two independent *SOX9* deleted clones (average score 0.984 vs. 0.686; t test p value 0.033; Figure S3G). Gene set enrichment analysis (GSEA) further confirmed the enrichment of intestinal stem cell, *MYC* and oxidative phosphorylation expression programs in *SOX9* C-terminus deleted clones (Figure S3H). These findings suggest that *SOX9* mutations in IM may promote intestinal stem cell lineages and clonal expansion. However, the reduced frequency of *SOX9* mutations in GC suggests that *SOX9-*mutated IM clones may not be obligate precursors of malignancy.

### Spatiotemporal clonal dynamics in normal, IM, and dysplastic gastric tissues

**Figure 2:**
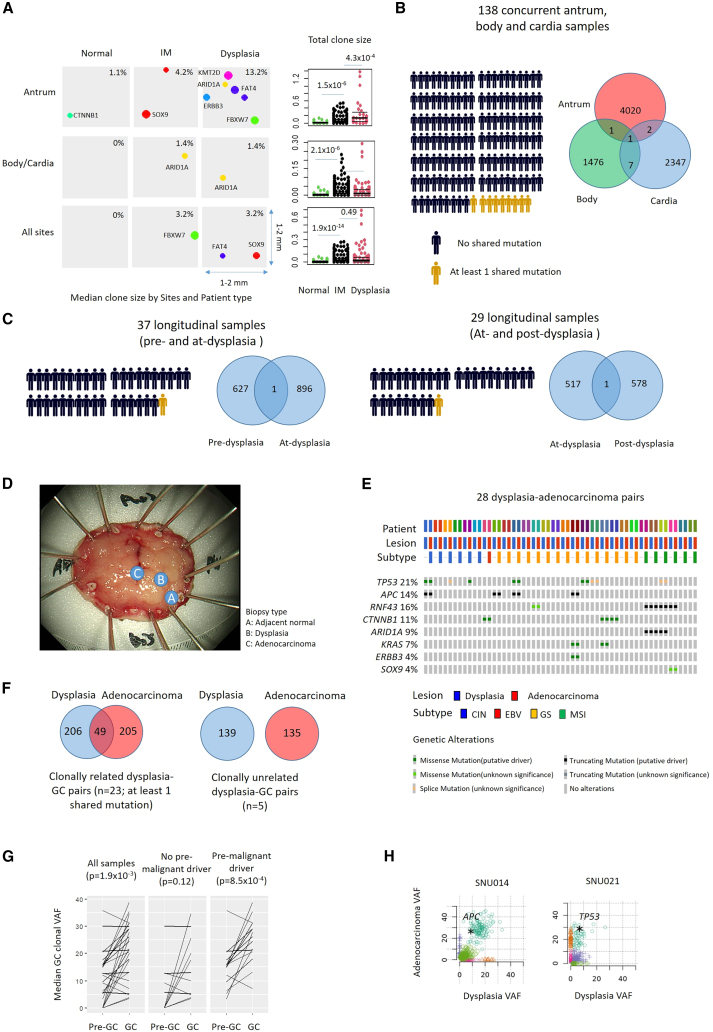
Clonal dynamics in IM, dysplasia, and early GC (A) Bubble plots showing predicted genetic clones in normal, IM, and dysplasia. Clone sizes were inferred from VAFs. Bubble sizes were plotted proportionally. Each driver mutation is represented with a distinct color. Each square represents the size of biopsy (1–4 mm^2^). Beeswarm plots show total clone sizes in normal, IM, and dysplasia samples by stomach region or across all regions. p values utilized Wilcoxon tests. (B) Shared (gold) and private (black) somatic mutations in pre-malignant samples from different stomach sites in the same subject (n = 138). Human silhouettes indicate the number of samples with shared somatic mutations in at least two sites. Venn diagrams indicate the number of shared and private somatic mutations in antrum, body, and cardia. (C) Shared (gold) and private (black) somatic mutations in longitudinal samples from the same subject, either (left) from pre-dysplasia to dysplasia (n = 37) or dysplasia to post-dysplasia (n = 29). Venn diagrams indicate the number of shared and private somatic mutations in pre-, at-, and post-dysplasia samples. (D) WES of samples exhibiting concurrent normal, dysplasia, and early GC. (E) Oncoplot showing selected GC driver genes in 28 dysplasia-early GC pairs. Many mutations in dysplasia are also observed in concurrent GC. (F) Sharing of mutations in clonally related (n = 23) and unrelated (n = 5) dysplastic-GC pairs. Median numbers of shared and private mutations in dysplasia and GC lesions are indicated. (G) Median clone sizes in dysplastic and GC samples, with or without driver mutations in the dysplastic lesion. p values utilized paired Wilcoxon tests. (H) SciClone 2D plot showing clonal expansions associated with selected driver genes (*APC* and *TP53*) in dysplasia and concurrent GC.

We compared genetically related cell populations (“clones”) between different categories of gastric pre-malignancy. Biopsies from IM subjects were often polyclonal (median clone size 3.2%) while similarly sized clones were rare in normal subjects (median size 0%; Wilcoxon test p value 1.9 × 10^−14^). Clone sizes expanded further in biopsies concurrent with dysplasia, particularly in the antrum (median size 13.2%; p value 4.3 × 10^−4^) but not body/cardia (median size 1.4%; p value 0.50) (Figure 2A), consistent with clinical observations where dysplastic and early GC lesions often emerge from the antrum.

To ask if IM clones are shared between different stomach regions in the same subject (“intra-subject”), we analyzed 115 IM subjects where multiple biopsies were sampled from different regions (antrum, body, and cardia) at the same time point (138 antral/body/cardia trios in total). Only 8 subjects (9 samples) had IMs from different regions sharing at least one mutation, with the vast majority of subjects exhibiting genetically unrelated clones (Figure 2B). Further, to ask if these clones are stable or fluctuate dynamically over time, we then analyzed 66 matched longitudinal pairs from the same subject, where IMs were sampled at different time points (37 pairs: pre-dysplasia to adjacent-to-dysplasia; 29 pairs: adjacent-to-dysplasia to subsequent dysplasia regression). Shared mutations were observed in only 2 subjects (3.0%), suggesting that most IM clones are dynamic and transient (Figure 2C).

We hypothesized that in contrast to IM where clones are transient, gastric clones exhibiting dysplasia might be more persistent contributing to their larger sizes. To explore this, we profiled 28 GC patients where in each patient normal gastric tissue, dysplastic tissue, and early GCs were concurrently sampled (Figure 2D). In the matched GC-dysplasia pairs, the majority of driver gene mutations (22/26) observed in the GCs were also observed in the patient-matched dysplasia (e.g., *TP53*, *APC*, *ARID1A*) (Figure 2E), with most pairs (23/28) showing at least one shared mutation between dysplastic lesions and matched GCs (Figure 2F). Clonal reconstructions confirmed clonal expansions from dysplastic (median clonal VAF 12.4%) to malignant GC (median clonal VAF 21.4%) (paired Wilcoxon test p value 1.9 × 10^−4^; 26 pairs), with more pronounced expansions in dysplastic lesions containing driver mutations (n = 15; paired Wilcoxon test, p value 8.5 × 10^−4^) (Figures 2G and 2H). These results suggest that in IM, independent clones can arise at different stomach sites, but the majority of these IM clones are likely transient possibly due to high turnover rates. In contrast, genetic clones in dysplastic tissues may be more persistent, increasing the likelihood of transforming to full malignancy.

### IM scRNA-seq reveals shifts in gastric tissue ecology with expansions of intestinal cell lineages

**Figure 3:**
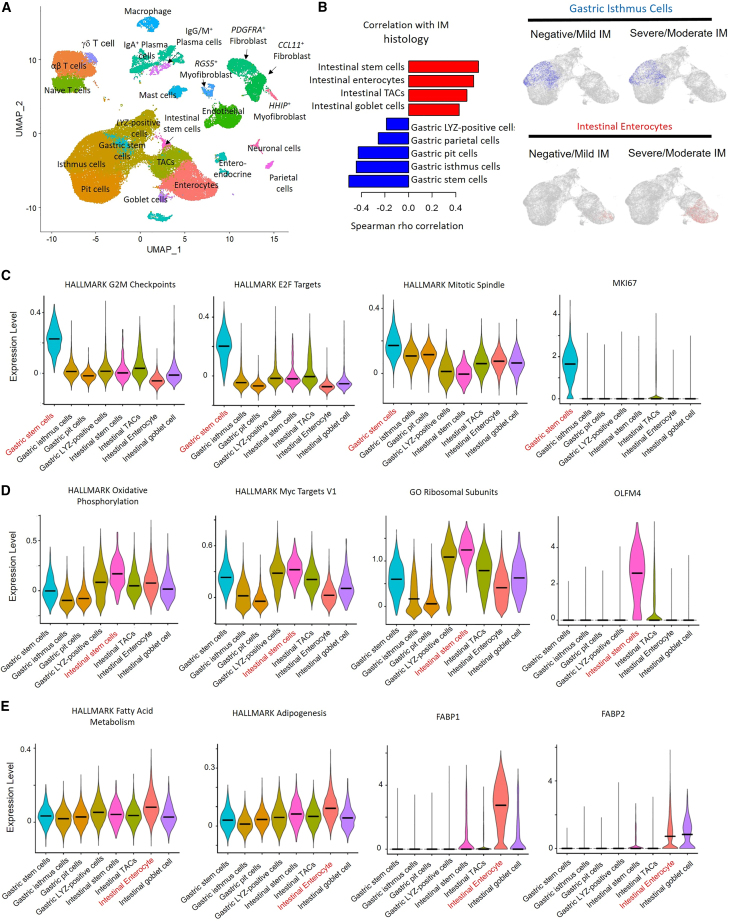
IM scRNA-seq landscape (A) Cell types/lineages identified from single-cell RNA-seq of antral IMs. (B) Barplot showing increasing intestinal lineage cell types and decreasing gastric lineage cell types between IM histological grades. Feature plots depict selected intestinal and gastric lineage cells in severe/moderate IM compared with mild/negative IM. (C) Violin plots showing enrichment of cell cycle pathways in gastric stem cell lineages. (D) Violin plots of oxidative phosphorylation and Myc target V1 pathways highlights expression in intestinal stem cell lineages. Also shown are *OLFM4* expression levels. (E) Violin plots showing enrichment of fatty acid metabolism and adipogenesis pathways in intestinal enterocyte lineages. Intestinal enterocytes are marked by expression of *FABP1* and *FABP2*. See also Figure S4.

To define the gamut of cellular lineages in IM, we performed scRNA-seq (single-cell RNA sequencing) of 18 antral IMs from non-cancer subjects exhibiting differing IM severity levels (5 negative; 7 mild; 4 moderate; 2 severe). Performing clustering on 71,933 cells, we identified 23 cell types and 4 major lineages, including gastric (40.9% of cells; marked by *TFF2*), intestinal (18.2%; *REG4*), immune (25.4%; *SRGN*), and stromal cells (12.6%; *DCN*) (Figures 3A and S4A). We observed four gastric lineages including gastric stem cells (*IQGAP3*, *STMN1*, and *MKI67*), isthmus cells (*SULT1C2*, *CAPN8*, and *TFF1*), LYZ-positive cells (*LYZ*, *MUC6*, and *PGC*^26^^,^^27^^,^^28^), and immature and mature pit cells (*GKN1*, *GKN2*, and *TFF2*) (Figure S4B). Similarly, we observed four intestinal-type lineages, including intestinal stem cells (*OLFM4* and *CDCA7*), transit amplifying cells (TAC; *DMBT1*), enterocytes (*FABP1*, *FABP2*, and *KRT20*), and goblet cells (*SPINK4*, *MUC2*, and *TFF3*) (Figure S4C). IM severity correlated with increased proportions of intestinal cell lineages (Spearman’s correlation, rho = 0.67, p value 2.1 × 10^−3^), including intestinal stem cells (rho = 0.55), TACs (rho = 0.50), and enterocytes (rho = 0.60), with decreases of gastric cell lineages (rho = −0.59, p value 9.7 × 10^−3^) (Figure 3B).

Gastric stem cells (*IQGAP3*) exhibited upregulated pathways related to cell division (NES 3.3, adjusted p value 9.9 × 10^−28^), E2F targets (NES 3.4, adjusted p value 2.0 × 10^−31^), mitotic spindle (NES 2.9, adjusted p value 4.5 × 10^−14^), *MKI67* (90.3% vs. 4.8%; adjusted p value <2.2 × 10^−308^), and *TOP2A* expression (83.1% vs. 3.6%; adjusted p value < 2.2 × 10^−308^) (Figure 3C). Intestinal stem cells (*OLFM4*) were highly enriched in oxidative phosphorylation (NES 3.4; adjusted p value 1.9 × 10^−16^), MYC pathways (NES 3.3; adjusted p value 2.4 × 10^−14^), and ribosomal genes (Figure 3D). Compared to intestinal stem cells, intestinal enterocytes (which are more differentiated, *FABP1/2*) exhibited high expression of adipogenesis (NES 1.9; adjusted p value 4.6 × 10^−3^) and fatty acid metabolism programs (NES 1.7; adjusted p value 2.5 × 10^−2^) and MYC pathway down-regulation (NES -2.9, adjusted p value 1.3 × 10^−9^) (Figure 3E). Notably, *SOX9* was highly expressed in gastric LYZ-positive cells (56.8%, adjusted p value < 2.2 × 10^−308^) and intestinal stem cells (29.9%, adjusted p value 7.9 × 10^−28^), with intestinal stem cells expressing high levels of *SOX9*-associated signatures (e.g., stemness, oxidative phosphorylation, and MYC targets).

We also analyzed immune and stromal cell types. The immune cells clustered into naive T cells (*PTPRC*^+^ and *CD52*^−^), mature αβ T cells (*PTPRC*, *CD52*, and *CD8A*), γδ T cells (*TRDC* and *TRGC1*), IgA^+^ plasma (*MZB1*, *IGHA1*, and *IGHA2*) and IgG/M^+^ plasma cells (*MZB1*, *IGHG1*, and *IGHM*), mast cells (*TPSB2*), and macrophages (*CD14* and *FCGR2A*) (Figure S4D). We also identified stromal cells corresponding to endothelial (*PLVAP* and *FLT1*), *CCL11*^+^ (*CCL11*, *ABCA8*, and *LUM*) and *PDGFRA*^+^ (*PLAT*, *POSTN*, and *PDGFRA*) fibroblasts, and *RGS5*^+^ (*RGS5*, *CD36*, and *PDGFRB*) and *HHIP*^+^ (*HHIP*, *ACTA2*, and *TAGLN*) myofibroblasts (Figure S4E). When correlated to the proportion of intestinal lineages (intestinal stem cells, TACs, goblet, and enterocytes), we observed a positive correlation between γδ T cells with intestinal-type cells as previously reported^29^ (rho 0.33, p value 0.18). (Figure S4F). Compared to mature αβ T cells, γδ T cells expressed higher levels of markers associated with immune exhaustion such as *ENTPD1* (57.9% vs. 3.9%, adjusted p value < 2.2 × 10^−308^), *TIGIT* (49.0% vs. 22.6%; adjusted p value 4.7 × 10^−51^), and *HAVCR2* (25.1% vs. 4.4%; adjusted p value 3.8 × 10^−94^) (Figure S4G). Consistent with this, GSEA analysis applied to the γδ T cells revealed down-regulation of the TNFα signaling pathway in severe/moderate IM patients compared to mild/negative IMs (NES -2.3, adjusted p value 6.1 × 10^−8^) (Figure S4H).

### Intestinal stem-cell dominant IM exhibits transcriptional similarities to GC

**Figure 4:**
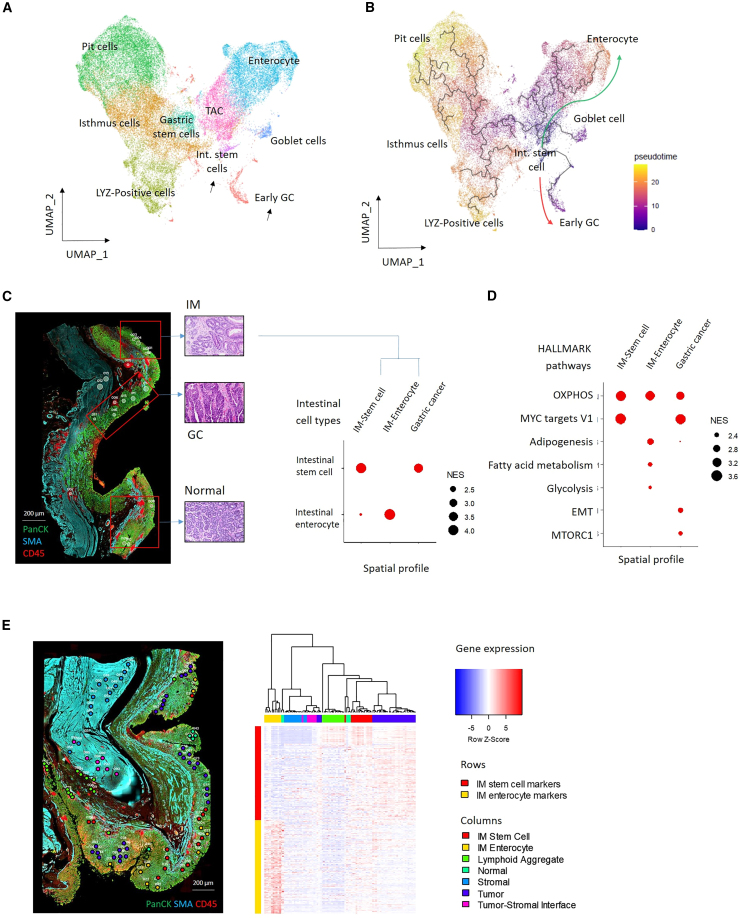
Trajectory analysis of IM and GC cells (A) UMAP projection using gastric and intestinal cell types in IM and early GC cells obtained from Kumar et al.^30^ GC cells and intestinal stem cells are marked by black arrows. (B) Monocle3 trajectory analysis. GC cells are most closely related to intestinal stem cells. (Red arrow) Differentiation path from intestinal stem cells to early GC. (Green arrow) Differentiation path from intestinal stem cells to enterocytes. (C) Representative ROIs from a tissue section displaying concurrent normal, IM, and GC (left). AOIs/ROIs from IMs were annotated as stem cells dominant IM (IM-stem cell) or enterocyte dominant (IM-Enterocyte) based on scRNA-seq profiles (right). NES values utilized fgsea. Scale bar, 200 μm. (D) Dotplots showing enrichment of selected HALLMARK pathways in intestinal stem cell dominant IM, enterocyte-dominant IM, and GC. GCs also exhibit EMT and MTORC1 signatures. NES values utilized fgsea. (E) Image of histological slide with selected ROIs (left). IM regions were annotated as intestinal stem cell-dominant or enterocyte-dominant IM. Hierarchical clustering using IM stem cell and enterocyte markers of selected ROIs demonstrates similarities between GC and intestinal stem-cell dominant IM (right). Scale bar, 200 μm. See also Figure S5.

To investigate relationships between the IM gastric and intestinal lineages with malignant GC, we integrated the IM scRNA-seq data with previously published scRNA-seq data from early stage GCs (GC scRNA-data was restricted to epithelial cells exhibiting inferred sCNAs)^30^ (Figure S5A). Clustering of the combined IM and GC data confirmed close similarities between IM and GC epithelial cell populations (Figure 4A). Monocle3 trajectory analysis projected that early GC cells appear to be most closely related to intestinal stem-cell lineages, and more distantly related to other intestinal-related lineages such as differentiated enterocytes (Figure 4B). These findings suggest that IM intestinal stem-cell subpopulations may harbor a potential cellular reservoir for the emergence of intestinal-type GC. Indeed, some *OLFM4*-expressing intestinal stem cells also co-expressed *LGR5* (85/296 cells; 28.7%; Fisher’s test OR 2.8, p value 0.021) and *AQP5* (151/296 cells; 51.0%; Fisher’s test OR 2.5, p value 5.1 × 10^−3^), both previously proposed to mark GC stem cells.^31^

To orthogonally confirm that IM intestinal stem-cell lineages are related to GC, we performed spatial transcriptomics on tissue sections from eight GC patients harboring concurrent normal, IM and GC regions. Across 87 IM AOIs/ROIs (areas-of-illumination/regions-of-interest), we calculated enrichment scores to annotate each IM region as “stem-cell dominant (n = 37)” or “enterocyte-dominant (n = 30)” using the scRNA-seq expression signatures (Figure 4C). Biological pathways activated in stem cell-dominant IM, enterocyte-dominant IM and GC were further inferred using HALLMARK.^32^ Consistent with the scRNA-seq data, stem-cell dominant IMs overexpressed oxidative phosphorylation gene sets (NES 3.5, adjusted p value 1.4 × 10^−40^) and MYC target pathways (NES 3.7, adjusted p value 4.2 × 10^−49^), which were notably also expressed in GC regions (Oxidative phosphorylation - NES 3.1, adjusted p value 1.1 × 10^−27^; MYC - NES 3.5, adjusted p value 2.2 × 10^−44^) (Figure 4D). In contrast, pathways specific to enterocyte-dominant IM included fatty acid metabolism (NES 2.4, adjusted p value 1.1 × 10^−7^), and adipogenesis (NES 2.7, adjusted p value 1.2 × 10^−12^) which were not strongly up-regulated in GC (Fatty acid metabolism - NES 1.5, adjusted p value 0.038; adipogenesis - NES 2.1, adjusted p value 5.3 × 10^−7^). Compared to both stem-cell dominant and enterocyte-dominant IM, GC regions harbored additional signatures not observed in IMs such as epithelial-mesenchymal transition (EMT, NES 2.4, adjusted p value 4.6 × 10^−11^) and MTORC1 signaling (NES 2.3, adjusted p value 2.2 × 10^−9^). To illustrate, hierarchical clustering of the spatial transcriptomics data from a single slide (93 ROIs) clustered stem cell-dominant IMs together with GC while enterocyte-dominant IMs were more distantly related (Figure 4E). These results demonstrate that even in the same subject, IMs display significant lineage heterogeneity with stem-cell dominant IM exhibiting expression signatures similar to malignant GC.

Interestingly, we observed a significant negative correlation between stem-cell dominant IM and inflammatory pathways (rho −0.45, p value 7.0 × 10^−4^) while enterocyte-dominant IM was positively associated with inflammation (rho 0.63, p value 6.8 × 10^−7^) suggesting that IM stem cells may occupy an immune-excluded niche (Figure S5B). In enterocyte-dominant IM regions, we observed high expression of immune-related genes involved in antigen presentation (*B2M*, *HLA-A*, *HLA-B*, and *HLA-C*) and an enrichment of activated immune pathways by GSEA (Figure S5C). In contrast, stem-cell dominant IM regions were enriched with Myc-target pathways, lacking immune gene/pathway expression signatures (Figure S5D), reminiscent of observations in gastric metaplasia of the colon where stem cell regions are immune cold.^33^ To explore this immune suppression, we further analyzed scRNA-seq data to assess relationships between IM stem cells with immune cell types. We observed positive correlations between IM stem cells with a γδ T cell (*TRDC* and *TRGC1*) (rho 0.53, p value 0.024) subpopulation overexpressing canonical markers of T cell exhaustion including *ENTPD1* (57.9% vs. 8.6%; adjusted p value <2.2 × 10^−308^), *TIGIT* (49.0% vs. 2.9%; adjusted p value <2.2 × 10^−308^), and *HAVCR2* (25.1% vs. 3.6%; adjusted p value 3.4 × 10^−193^). *ENTPD1/*CD39-positive γδ T cells are a subclass of γδ T-cells reported to promote immunosuppression via the adenosine pathway.^34^^,^^35^^,^^36^ These results represent plausible contributors to the reduced immune activity in IM stem cell-dominant regions.

### Bulk transcriptome sequencing of IM identifies distinct expression subtypes

**Figure 5:**
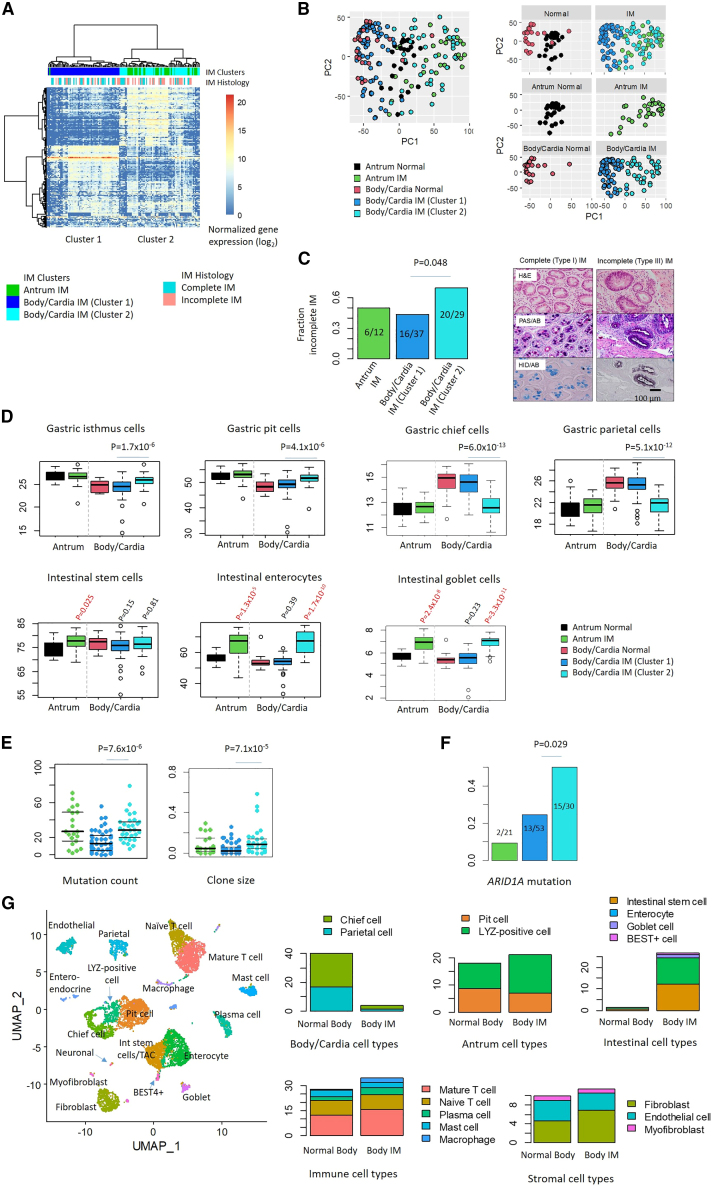
Expression-based molecular subtypes of IM and pseudoantralization (A) Hierarchical clustering of bulk IM RNA-seq transcriptomes (n = 137 IM). A cluster of body/cardia IMs (cluster 2, light blue) cluster with antral IMs (green). (B) PCA graphs of normal gastric samples and IMs. Normal antral and body/cardia samples were well demarcated, while IM samples are distributed across both regions. Pseudoantralized IMs cluster with antral IMs. (C) Fraction of histologically defined incomplete and complete IM subtypes across IM expression subtypes (left). p values utilized Fisher’s test. Representative images of Type I complete and Type III incomplete IM (right; adapted from Huang et al.^11^). Scale bar, 100 μm. (D) Single sample GSEA (ssGSEA) scores for gastric cell types and intestinal cell types in antral and body/cardia normal samples and IMs. Pseudoantralized IMs exhibit similarities to antral IMs. p values were estimated using Wilcoxon tests. Box, median +/− IQR. Whiskers, 1.5× IQR**.** (E) Mutation counts and clone sizes of IM expression subtypes. Pseudoantralized IMs exhibit higher mutation counts and clone sizes relative to Cluster 1 body/cardia IMs. p values utilized Wilcoxon tests. (F) *ARID1A* mutations are enriched in pseudoantralized IMs. p values utilized Fisher’s tests. (G) Proportion of cell types from scRNA-seq of gastric body biopsies (n = 6). See also Figure S6.

To ask if IMs can be classified into distinct expression-based molecular subtypes^37^^,^^38^, we analyzed bulk RNA-seq transcriptomes of 183 gastric samples from the antrum (24 normal, 31 IM) and body/cardia (22 normal, 106 IM). Expression-based clustering of the normal gastric samples confirmed a distinct separation of antral and body/cardia samples (Figure 5A). We then overlaid the IM gene expression data to reveal three distinct IM subtypes. The first IM subtype comprised antral IMs with expression similarities to antral gastric tissues (30/31), while the second subtype comprised body/cardia IMs with expression similarities to body/cardia normal tissues (64/106). However, we noted a third subtype comprising IMs from the stomach body/cardia but expressing transcriptional similarities with antral IMs, referred to as “pseudoantralized IMs” (42/106, Figure 5B). This phenomenon is reminiscent of “pseudoantralization”, a process characterized by the appearance of antral-type mucosa in the body/cardia and associated with Hp infection, IM, and GC.^39^

Several lines of evidence support pseudoantralized IMs as a distinct molecular entity. First, when correlated to histology, pseudoantralized IMs were moderately associated with incomplete IM histology (Figure 5C; Fisher’s test p value 0.048), a histological subtype associated with higher GC risk.^40^ Second, compared to body/cardia IMs, pseudoantralized IMs harbored increased gene expression programs of antral cell types (gastric pit; Wilcoxon test p value 4.1 × 10^−6^ and isthmus cells; p value 1.7 × 10^−6^), and mature intestinal cell lineages (enterocytes; p values 1.7 × 10^−10^ and goblet cells; p values 3.3 × 10^−11^), with reduced expression of body/cardia cell types (gastric chief; p value 6.0 × 10^−13^ and parietal cells; p value 5.1 × 10^−12^) (Figure 5D). Third, pseudoantralized IMs exhibited significantly higher mutation rates (Wilcoxon test p value 7.6 × 10^−6^) and clone sizes (p value 7.1 × 10^−5^) compared to body/cardia IMs and similar to antral IMs (Figure 5E). Fourth, pseudoantralized IMs exhibited a higher frequency of *ARID1A* mutations compared to antral IMs (Fisher’s test p value 0.0028) or body/cardia IMs (Fisher’s test p value 0.029) (Figure 5F). Pseudoantralized IMs exhibited features reminiscent of SPEM including higher expression of *TACSTD2* (encoding TROP2, a marker for incomplete IM^41^; log2FoldChange 1.8 compared to normal body/cardia; DESeq2 adjusted p value 5.0 × 10^−5^).

We also performed scRNA-seq on 6 gastric body biopsies (4 IMs and 2 normal; Figures 5G and S6A), identifying 18 cell clusters including gastric body lineages (chief and parietal cells), antral lineages (LYZ-positive cells and pit cells), and intestinal lineages (intestinal stem cell, goblet cells, and enterocytes) (Figures S6B and S6C). Compared to normal body samples, body IMs exhibited depletion of normal body cell types (3.8% vs. 40.0%) and an increase in intestinal cell types (26.6% vs. 1.3%) (Figure 5G). Compared to antrum IMs, body IMs exhibited lower proportions of gastric cell types (24.8% vs. 37.4%) and higher immune cells (34.8% vs. 25.5%), while intestinal (25.9% vs. 22.5%) and stromal (11.3% vs. 11.5%) cell type proportions were similar (Figure S6D).

### Pseudoantralized IMs exhibit an inflammatory microenvironment associated with a distinctive oral microbial community

**Figure 6:**
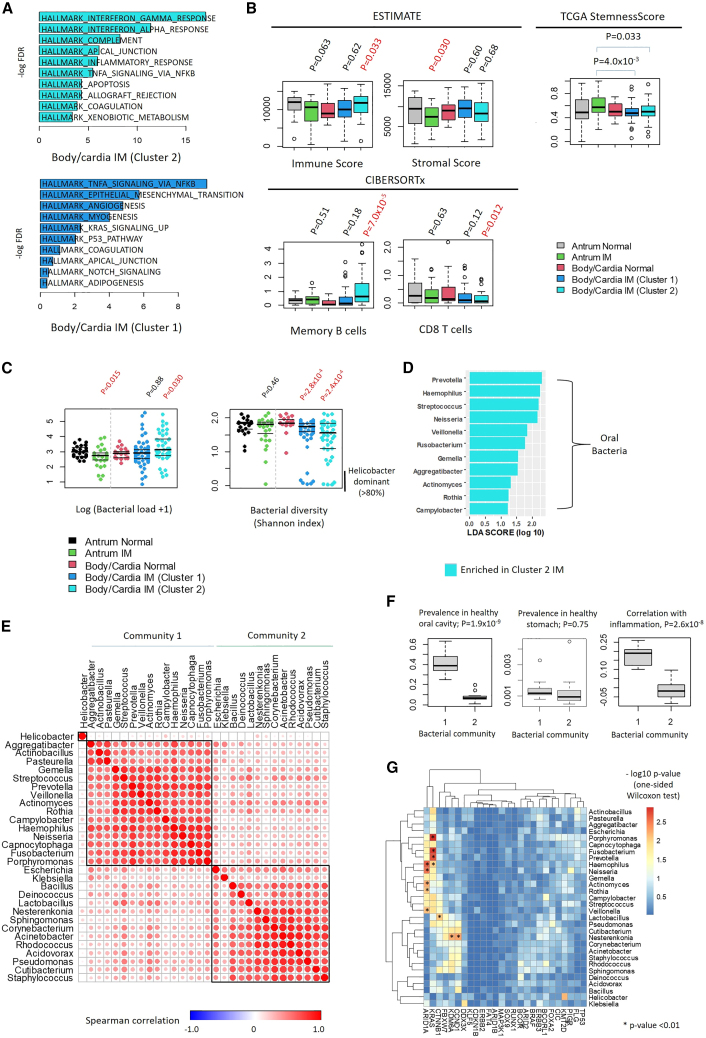
Immune landscape in IM (A) GSEA of expression signatures in IM subtypes. Inflammatory signatures (Interferon gamma, etc) are upregulated in subtype 2 (Pseudoantralized IM). (B) Immune, stromal, and stemness content deconvolution analysis using ESTIMATE, CIBERSORTx, and TCGA stemnessScore. Pseudoantralized IMs exhibit upregulation of immune scores and B cell programs while antral IMs show higher stem cell features. p values utilized Wilcoxon tests. Box, median +/− IQR. Whiskers, 1.5× IQR. (C) Bacterial density and diversity in IM and normal samples. Pseudoantralized IMs exhibit increased bacterial loads but lower diversity. p values utilized Wilcoxon tests. (D) LDA analysis comparing microbial genera between body/cardia IM subtypes. LDA effect sizes utilized lefser. (E) Spearman analysis of the 30 most abundant bacterial genera, representing the major contributors to microbial levels in this study. Two distinct microbial communities are observed (C1 and C2). (F) Prevalence of C1 and C2 communities in reference microbiomes from oral cavity (left) and normal stomach (middle). Correlation between community C1 with HALLMARK inflammation scores (right). p values utilized Wilcoxon tests. Box, median +/− IQR. Whiskers, 1.5× IQR. (G) Association between bacterial genus abundance with IM driver mutations. Bacterial genera positively associated with somatic mutations are indicated with asterisks (p < 0.01). See also Figure S7.

Pathway analysis of the bulk IM RNA-seq profiles revealed that both pseudoantralized IMs and body/cardia IMs exhibited increased TNFα signaling pathway expression (pseudoantralized IM-NES 2.0, adjusted p value 1.3 × 10^−5^; body/cardia IM - NES 2.3, adjusted p value 4.7 × 10^−8^) (Figure 6A). This is consistent with scRNA-seq data showing a higher proportion of immune cell types in body IMs relative to antral IMs. Pseudoantralized IMs exhibited increased interferon-α (NES 2.6; adjusted p value 3.9 × 10^−12^) and interferon-γ responses (NES 2.7; adjusted p value 9.3 × 10^−18^) exceeding native body/cardia IMs. Using CIBERSORTx^42^ and ESTIMATE,^43^ we confirmed significant increases of immune cells in pseudoantralized IMs (Figure 6B, Wilcoxon test p value 0.021 in pseudoantralized IM) largely associated with memory B cells (Wilcoxon test p value 5.6 × 10^−5^). Compared to antral IMs which exhibit both enterocyte-dominant (immune activated) and stem-cell dominant (immune-suppressed) populations (Figure 5D), pseudoantralized IMs exhibited reduced stem-cell dominant features (Figure 6B) which may result in their behaving more similarly to immune-active enterocyte-dominant IM regions with activated immune pathways.

We hypothesized that alterations in microbial composition might contribute to the pseudoantralized IM inflammatory microenvironment. Using PathSeq^44^, we estimated bacterial content and diversity from the RNA-seq data which enables the identification of transcriptionally active bacterial communities at the genus level.^45^ We identified ∼34 million bacterial reads from 847 bacterial genera in the 183 samples. *Helicobacter* sequences accounted for 79.3% of all unambiguously mapped bacterial reads. High *Helicobacter* levels were found in 8.8% of IM samples and correlated significantly with histology (Fisher’s test, p value 1.7 × 10^−12^, OR 458.3) (Figures S7A and S7B). Compared to body/cardia normal samples, pseudoantralized IMs exhibited both increased bacterial levels (Wilcoxon test p value 0.030) and reduced biodiversity (p values 2.8 × 10^−4^ in non-antralized IM, 2.4 × 10^−4^ in pseudoantralized IM) (Figure 6C). The combination of increased bacterial load with decreased diversity (sometimes termed “microbial dysbiosis”) has been linked to various diseases such as rheumatoid arthritis^46^ and diabetes.^47^

Linear discriminant analysis (LDA) highlighted bacterial communities comprising *Streptococcus*, *Prevotella*, and *Fusobacterium* in pseudoantralized IM (LDA score 1 to 3) compared to non-antralized IM (Figure 6D). A more refined clustering of the top 30 most abundant bacterial genera yielded two clusters of bacterial communities (Figure 6E). Cluster 1 comprised bacteria normally associated with the oral cavity (e.g., *Streptococcus*, *Porphyromonas*) (Wilcoxon test p value 1.9 × 10^−9^) but typically absent in healthy stomach (p value 0.75). These observations recall previous reports that certain oral bacteria may be associated with IM onset after *H. pylori* eradication.^48^ We confirmed that our cluster 1 community overlapped significantly with these previous reports (Fisher’s test p value 6.3 × 10^−3^). Notably, levels of oral microbes were also significantly associated with inflammation (Figure 6F; p value 2.6 × 10^−8^) suggesting that the presence of these microbes may be pro-inflammatory, and also with driver gene mutations such as *ARID1A* and *KRAS* (Figure 6G). To assess the persistence of these oral microbes through dysplasia and GC, we performed bulk RNA-seq on the matched adjacent non-malignant/dysplasia/GC samples (Figure 2D; for RNA-seq 68 samples; 22 non-malignant, 23 dysplasia, and 23 early GC). Oral bacterial abundances were marginally higher in adjacent non-malignant gastric tissues compared to paired dysplastic samples (Figure S7C; Wilcoxon test, p value 0.030). Notably, we observed substantial levels of oral bacteria persisting in both dysplastic and GC samples, which were significantly associated with immune pathway activation (Figures S7D and S7E). These observations suggest that oral bacterial infection occurs prior to dysplasia, persists through dysplasia and GC, and is associated with persistent inflammation.

### Combined genomic-clinical predictive models outperform models based on clinical information only

**Figure 7:**
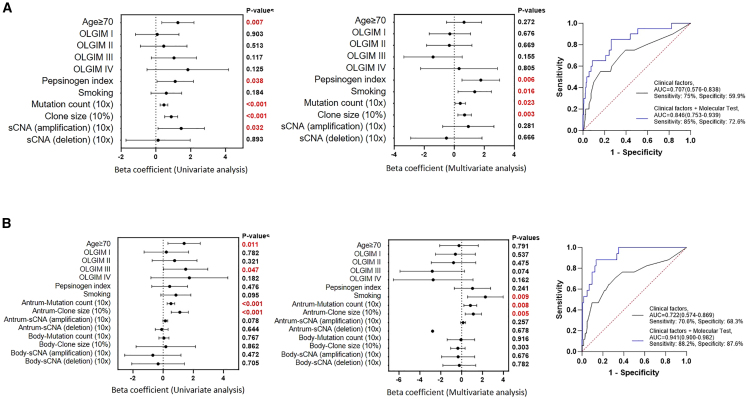
Predicting IM progression risk from clinical and genomic features (A) Clinical factors (age≥70, operating link for gastric intestinal metaplasia (OLGIM) score, pepsinogen index, smoking status) and genomic features (mutation count, clone size, sCNA (amplification/deletion) used to stratify gastric dysplasia risk in antral biopsies. p values utilized logistic regression. (Right) Receiver operating characteristic (ROC) curve showing accuracy of prediction based on clinical factors only (gray) or clinical and genomic factors (blue). (B) Analysis of patients with both antral and body biopsies (Dysplasia n = 20 vs. Non-dysplasia n = 186). Left panel shows forest plots of univariate and multivariate logistic regression analysis. The right panel shows ROC curves and corresponding AUC values to evaluate model performance. See also Figure S8 and Table S4.

Finally, we evaluated if combining genomic information with clinical data might improve current clinical models used to stratify IM patients for dysplasia risk.^14^ First, we focused on antral samples comparing genomic and clinical features at the time of dysplasia to non-dysplasia subjects (Figure 7A). We found that having a positive pepsinogen index (B = 1.768, 95% CI (confidence interval) 0.519–3.017, p = 0.006), smoking history (B = 1.363, 95% CI 0.249–2.477, p = 0.016), higher mutation counts (B = 0.04, 95% CI 0.005–0.075, p = 0.023), and larger clone sizes (B = 6.88, 95% CI 2.386–11.374, p = 0.003) significantly increased the risk of dysplasia. Notably, combined molecular and clinical models achieved superior performance in predicting dysplasia (Area under the curve; AUC = 0.846, 95% CI 0.753–0.939, p < 0.001, sensitivity 85%, specificity 72.6%) compared to clinical models alone (AUC = 0.707, 95%CI 0.576–0.838, p = 0.002, sensitivity 75%, specificity 59.9%). Incorporation of microbial data (total bacterial load, oral bacterial levels, gastric bacterial levels, or HP abundance) did not improve model performance (Figures S8A and S8B). We performed cross-validation analysis by splitting the current cohort equally into two random parts and repeating the randomization and measurements (Figure S8C). Similar to the overall cohort, integrated molecular and clinical models achieved superior performance compared to clinical models alone (Figure S8D) in discriminating dysplasia from non-dysplasia subjects with AUC values of 0.787 (sensitivity 84%, specificity 77%) in the discovery set and AUC 0.742 (sensitivity 85%, specificity 85%) in the validation set.

We conducted univariate and multivariate logistic regression to test if the 26 driver genes, or combinations of driver genes occurring in >1% of IM samples (45 combinations), are associated with dysplasia risk. Univariate analysis revealed that four individual genes and five gene-gene combinations were associated with dysplasia (p < 0.05). In multivariate analysis, three genes (*BRAF*, *BCORL1*, and *DDX3X*) and the combination of *ARID1A*/*ERBB3* mutations were associated with increased dysplasia risk (p < 0.05) (only factors with p < 0.15 in univariate analysis were assessed by multivariate analysis) (p < 0.05; Table S4). It is possible that some of these gene-gene combinations may reflect functional relationships (e.g., *FBXW7/KMT2D*), as shown by recent reports that *FBXW7* targets *KMT2D* for degradation.^49^

As IM in the stomach body may represent a more advanced pathology, we further interrogated the cohort considering both the antrum and body at the time of dysplasia (Figure 7B). Prediction accuracies of integrated molecular and clinical models (AUC = 0.941, 95% CI 0.9–0.982, p < 0.001, sensitivity 88.2%, specificity 87.6%) were higher compared to clinical models (AUC = 0.722, 95% CI 0.574–0.869, p = 0.003, sensitivity 70.6%, specificity 68.3%). These observations suggest that integrating molecular information with clinical data is likely to improve prediction models stratifying the GC risk of subjects with gastric pre-malignancy.

## Discussion

To our knowledge, the present study reports the largest genomic and transcriptional survey of human IMs from a longitudinal prospective cohort. Our results indicate that IMs are not a homogeneous entity but highly heterogeneous between and within patients. Histologically, IMs can be classified into complete or incomplete subtypes,^50^ and a meta-analysis reported that incomplete IMs (pooled OR 9.48) were significantly associated with GC compared to complete IMs (pooled OR 1.55).^51^ GC onset was also higher among patients with IM involving the antrum and body (extensive IM; pooled OR = 7.39) compared to the antrum only (pooled OR = 4.06).^51^ These differences may be contributed at least in part by region-specific cellular populations in the stomach including stem cells. For example, antral isthmus stem cells are a potential stem cell population with high proliferative potential,^52^ and LGR5/AQP5-expressing stem cells in the antral gland base have also been identified as a potential source of IM and GC.^31^^,^^53^ In the gastric body, lineage tracing has revealed that chief cells can undergo transdifferentiation into SPEM,^54^ which is also associated with GC.^55^

Advances in sequencing have enabled the study of mutated genetic clones (genetically identical subpopulations of cells) and subclones in normal, inflamed, and pre-malignant tissues.^56^ Here, *SOX9* was identified as a new IM driver gene exhibiting enrichment in antral IMs. We found that *SOX9* mutations were associated with increased *SOX9* expression in primary GCs and functionally validated this phenomenon *in vitro*. Consistent with *SOX9*’s role as an intestinal stem cell factor,^23^ we observed increased intestinal stem cell proportions in antral IMs by bulk RNA-seq. This was supported by single-cell and spatial transcriptomics analysis confirming enrichment of Myc pathways in IM stem-cell dominant samples, which was also associated with a putative immunosuppressive environment characterized by γδ T-cells expressing exhaustion markers including *TIGIT*, *HAVCR2*, and *ENTPD1* (rho = 0.54, p = 0.024). In CRC, *SOX9* is mutated in 29% of cases^16^ with most *SOX9* alterations being nonsense/frameshift mutations preferentially clustering within the C-terminus^16^ and leading to *SOX9* overexpression.^25^ In CRC lines, *SOX9* overexpression led to reduced differentiation marker expression consistent with *SOX9* blocking intestinal differentiation in CRC. The overlap of *SOX9* mutational profiles between CRC and IM suggests that *SOX9* mutations may also play an initiating role in IM, by impeding differentiation and promoting lineage transformations and stem-like states.

However, while *SOX9* may promote IM clonal expansion, the lower frequency of *SOX9* mutations in GC suggests that not all *SOX9*-expanded IM clones may lead to cancer. One possible explanation might be that IM clones are dynamic and transient, in contrast to dysplastic clones that are larger and more stable with a higher propensity to transmit oncogenic genetic alterations to eventual GCs. Recent studies of pre-malignant tissues have revealed intriguing differences in mutated genes driving expansion in non-cancerous and cancerous tissues. For example, while *NOTCH1* mutations are a strong driver of clonal expansion in normal esophagus,^57^^,^^58^ these same mutations also impair tumor growth in mice models.^59^ These differences may be due to pre-malignant tissues experiencing distinct selective pressures from those related to cancer development.

Complementing bulk analysis, single-cell approaches are providing important insights into the lineage heterogeneities of metaplastic cells in the esophagus,^60^ stomach antrum,^27^ and colon.^33^ These studies have shown that Barrett’s esophagus (BE) may originate from normal gastric cardia tissues, and that esophageal adenocarcinomas (EAC) likely arise from a subset of undifferentiated BE cells expressing both intestinal and stem cell markers.^60^ In our study, we identified a subgroup of IM cells marked by expression of genes normally expressed in intestinal stem cells (*OLFM4*) (“intestinal stem-cell dominant”) and another IM subgroup displaying a more differentiated enterocyte phenotype. Single-cell and spatial analysis supports a close relationship between “intestinal stem-cell dominant” IM cells and early GC. We propose that similar to BE and EAC, gastric IMs with a higher proportion of intestinal stem-cell dominant IM lineages may be more undifferentiated and harbor a cellular reservoir for the eventual emergence of GC.

One notable finding was the identification of a distinct expression-based subtype of body-resident IMs exhibiting “pseudoantralization”. Pseudoantralized IMs exhibited depletions in body/cardia cell types and increased proportions of antral cell lineages. When contextualized against the existing literature, pseudoantralized IMs appear to exhibit many previously described features of SPEM, where aberrant antral type glands form in the stomach body due to parietal cell loss^9^ and chief cell transdifferentiation.^54^ We also found that pseudoantralized IMs exhibited pronounced inflammatory signatures, potentially implicating chronic inflammation in the pathogenesis of this particular IM subtype. Notably, by analyzing IM transcriptomes for microbial sequence reads, we discovered that pseudoantralized IMs exhibited increased bacterial levels compounded with reduced diversity, a hallmark of microbial dysbiosis.^61^ Intriguingly, pseudoantralized IMs were associated with a specific community of microbes normally associated with the healthy oral tract such as *Peptostreptococcus*, *Streptococcus*, and *Prevotella*. Lending credence to our results, it is worth noting that the oral microbes identified in our study displayed a strong overlap with IM-associated communities defined by 16S-based sequencing approaches.^48^ A role for microbial dysbiosis in IM development may suggest potential interventions for inhibiting the progression of pseudoantralized IMs through tailored antibiotics or improved oral hygiene.

**Figure 8:**
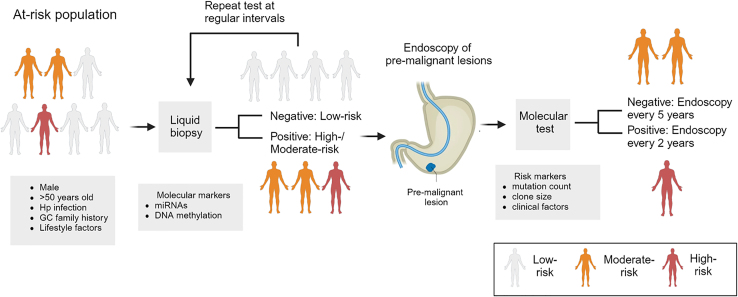
Precision prevention strategies for GC Surveillance of patients with pre-malignant conditions, such as intestinal metaplasia, using molecular tests assessing mutation load and genetic clones may be useful in stratifying “very-high-risk” individuals for endoscopic follow-up. Figure was created using Biorender software and based on Yeoh and Tan (2022).^68^

Our findings may have relevance for the management of patients with pre-malignant gastric lesions. Unlike countries such as Japan and South Korea where GC incidence is sufficiently high to warrant unselected population-based screening, mass population screening is not cost-effective in countries where GC incidence is moderate such as Singapore.^4^ As an alternative, applying differentiated screening approaches to patients stratified by distinct patterns of GC risk may represent a more sustainable strategy. Encouragingly, our results revealed that integrating genomic data into clinical risk stratification model improved risk model accuracy, suggesting the potential utility of genomic testing to identify individuals at very high risk of developing GC. Figure 8 proposes a potential clinical pathway for GC precision prevention, where subjects are first risk-stratified by either clinical criteria or inexpensive non-invasive assays (e.g., blood tests), and those deemed to be high risk are then offered more expensive endoscopic screening and molecular testing. Such a strategy may balance the tension between surveying large patient populations with the resource-intensive investments required for endoscopic procedures and advanced diagnostic testing including genomic sequencing.

### Limitations of the study

Our study has limitations. Rather than employing WES, our study used targeted DNA sequencing panels. Using a targeted panel was necessary to achieve high-sequencing depth (>1000×) to detect small clones harboring mutations in IM, and similar approaches have been previously used.^19^^,^^20^^,^^57^^,^^62^ However, one downside of a targeted panel is the possibility of missing important genes not covered on the panel. Our study also chiefly used Mutect2 as the main variant caller due to its sensitivity in detecting low VAF (<1%) somatic mutations.^63^^,^^64^^,^^65^ While low VAF mutations are more challenging to experimentally validate, our data suggests that a substantial number of these mutations are of functional significance, through integration of existing databases (STAR methods), exhibition of mutational signatures similar to higher VAF mutations (Figures S1E and S1F), and being rarely identified in control blood samples subjected to the same sequencing and analysis pipeline (STAR methods). A third limitation was that while we were able to validate the presence of several IM driver genes in an independent cohort, the current lack of other large-scale IM genomic datasets with mature follow-up information prevented us from independently validating the genome-clinical predictive models. While our ability to perform a robust internal cross-validation of the model provides some reassurance, a key goal will be to validate the predictive models in additional datasets as these become available. Finally, due to the predominance of intestinal-type GC cases from GCEP study, our study focus was primarily directed toward understanding the relationship between IM and intestinal GC. Further studies will be needed to determine if IM lineages might also be involved in the development of diffuse GC.^66^^,^^67^

## STAR★Methods

### Key resources table

**Table:** 

REAGENT or RESOURCE	SOURCE	IDENTIFIER
**Antibodies**		

SYTO13	Nanostring	GMX-RNA-MORPH-HST-12 #121300310; RRID: AB_2935724
PanCK	Nanostring	GMX-RNA-MORPH-HST-12 #121300310; RRID: AB_2935724
SMA	Nanostring	GMX-RNA-MORPH-HST-12 #121300310; RRID: AB_2935724
CD45	Thermo Fisher	# 50-9760-82; RRID: AB_2574362

**Biological samples**		

Gastric mucosa tissues (normal and IM patients)	This study	GCEP1000
Concurrent gastric cancer/dysplasia/adjacent normal	This study	Seoul National University
Gastric cancer samples	Xu et al.^24^	GASCAD

**Critical commercial assays**		

Wizard Genomic DNA Purification Kit	Promega	Cat# A1120
AllPrep DNA/RNA Micro Kit	Qiagen	Cat# 80284
QIAamp DNA Mini Kit	Qiagen	Cat# 51306
Qubit broad range assays	Thermo Fisher	Q32853
SureSelect Target Enrichment system - Agilent SureSelect XT HS2 DNA System with Pre-Capture Pooling with a customized tier 2 design	Agilent	G9985A, G9985B, G9985C, G9985D; Design ID: S3352052
NEBNext® Ultra™ II DNA Library Prep Kit	New England Biolabs	E7645
SMART-Seq Stranded Kit	Takara Bio	#634447
SureSelect XT HS2 DNA System with Pre-Capture Pooling	Agilent	G9985A, G9985B; 5190-8873
Chromium Single-Cell 3′ Library and Gel Bead Kit	10x Genomics	#1000190, #1000269
GeoMx Human Whole Transcriptome Atlas Human RNA for Illumina	Nanostring	GMX-RNA-NGS-HuWTA-4 #121401102
GeoMX Solid Tumor TME Morphology Kit	Nanostring	GMX-RNA-MORPH-HST-12 #121401102
GeoMx Seq Code Pack	Nanostring	GMX-NGS-SEQ-AB #121400201
T4 Polynucleotide Kinase	New England Biolabs	T M02015
BbsI	New England Biolabs	R0539S
BsaI-HF	New England Biolabs	R3733S
RNeasy Mini Kit	Qiagen	74106
GEL/PCR Purification Mini Kit	Favorgen	FAGCK 001
ReverTra Ace qPCR RT Master Mix with gDNA Remover	Toyobo	FSQ-301
Lipofectamine 3000 Transfection Kit	Invitrogen	L3000-015
KOD FX	Toyobo	KFX-101

**Deposited data**		

GCEP1000 DNA- and RNA-seq raw files	EGA	EGA: EGAS00001007067
Spatial transcriptomic raw files	EGA	EGA: EGAS00001007067
Single-cell RNAseq raw files	EGA	EGA: EGAS00001007067

**Experimental models: Cell lines**		

HFE145	Kind gift from Dr. Hassan Ashktorab, Howard University	–
GES-1	Kind gift from Dr. Alfred Cheng, Chinese University of Hong Kong	RRID:CVCL_EQ22
SNU-484	Korean Cell Line Bank	RRID:CVCL_0100

**Oligonucleotides**		

SOX9 Exon3 Genotyping Primer Forward	AAGTAGCAATTAGGTCTTCCGGACCC	This study
SOX9 Exon3 Genotyping Primer Reverse	ATCTACCTCCACGCTTGCTCTG	This study

**Recombinant DNA**		

pX330A-Cas9-2A-GFP-1x2	Kind gift from Dr. Shang Li, Duke-NUS Medical School, Singapore	–
pX330S-2	Kind gift from Dr. Shang Li, Duke-NUS Medical School, Singapore	–

**Software and algorithms**		

BWA-mem	Li et al.^68^	https://github.com/lh3/bwa
AGeNT	Agilent	https://www.agilent.com/en/product/next-generation-sequencing/hybridization-based-next-generation-sequencing-ngs/ngs-software/agent-232879
GATK	McKenna et al.^69^	https://gatk.broadinstitute.org/hc/en-us
Mutect2	Cibulskis et al.^72^	https://gatk.broadinstitute.org/hc/en-us/articles/360037593851-Mutect2
Varscan2	Koboldt et al.^73^	https://varscan.sourceforge.net/
Strelka2	Kim et al.^74^	https://github.com/Illumina/strelka
Funcotator	DePristo et al.^75^	https://gatk.broadinstitute.org/hc/en-us/articles/360035889931-Funcotator-Information-and-Tutorial
dNdSCV	Martincorena et al.^22^	https://github.com/im3sanger/dndscv
MutSigCV	Lawrence et al.^76^	https://www.genepattern.org/modules/docs/MutSigCV
Oncodrive-FML	Mularoni et al.^77^	https://bbglab.irbbarcelona.org/oncodrivefml/home
ASCAT	Van Loo et al.^79^	https://github.com/cancerit/ascatNgs
signature.tools.lib	Degasperi et al.^80^	https://github.com/Nik-Zainal-Group/signature.tools.lib
Hisat2	Kim et al.^81^	http://daehwankimlab.github.io/hisat2/
Stringtie	Pertea et al.^82^	https://ccb.jhu.edu/software/stringtie/
DESeq2	Love et al.^83^	https://bioconductor.org/packages/release/bioc/html/DESeq2.html
fgsea	–	https://bioconductor.org/packages/release/bioc/html/fgsea.html
PathSeq	Walker et al.^44^	http://software.broadinstitute.org/pathseq/
MSIsensor2	Niu et al.^86^	https://github.com/niu-lab/msisensor2
GISTIC2	Mermel et al.^87^	https://broadinstitute.github.io/gistic2/
Cell Ranger	10x Genomics	https://support.10xgenomics.com/single-cell-gene-expression/software/pipelines/latest/what-is-cell-ranger
Seurat	Hao et al.^88^	https://satijalab.org/seurat/
Monocles3	Cao et al.^92^	https://cole-trapnell-lab.github.io/monocle3/
CopyKAT	Gao et al.^90^	https://github.com/navinlabcode/copykat
phangorn	Schliep et al.^91^	https://cran.r-project.org/web/packages/phangorn/index.html
SpatialDecon	Danaher et al.^94^	https://bioconductor.org/packages/release/bioc/html/SpatialDecon.html
GeoMxTools	Nanostring	https://www.bioconductor.org/packages/release/bioc/html/GeomxTools.html
CIBERSORTx	Newman et al.^42^	https://cibersortx.stanford.edu/
ESTIMATE	Yoshihara et al.^43^	https://bioinformatics.mdanderson.org/estimate/index.html
SPSS	IBM	https://www.ibm.com/spss
GraphPad	Dotmatics	https://www.graphpad.com/features

### Resource availability

#### Lead contact

Further information and requests for resources and reagents should be directed to the lead contact, Prof Patrick Tan (gmstanp@duke-nus.edu.sg).

#### Materials availability

This study did not generate new unique reagents.

### Experimental model and subject details

#### Human subjects

Approvals were obtained from institutional review boards, including Domain Specific Review Board (DSRB) of the National Healthcare Group (2000/00329, 2019/00629), Centralized Institutional Review Board (CIRB) of Singapore Health Services (2018/3222), Institutional Review Board (IRB) of the National University of Singapore (LH-19-070E), IRB of Seoul National University Hospital (2005-053-1121), IRB of Yonsei University Wonju Severance Christian Hospital (CR319134), IRB of Nihon University School of Medicine (20191007) and Clinical Research Ethics Committee of Joint Chinese University of Hong Kong-New Territories East Cluster (2019.517). All study subjects provided informed consent prior to their participation in the studies.

At GCEP study conclusion, 82% of subjects had completed 5 years of follow-up, collectively representing 11157 person-years of surveillance.^14^ We observed development of 21 early gastric neoplasias, of which thirteen were high-grade dysplasia and eight Stage 1 GCs with the majority being intestinal-type (7 intestinal-type, 1 diffuse-type). The single diffuse-type GC was the only one of the eight GCs with no IM at baseline. Targeted DNA-seq (DNA-sequencing) and/or RNA-seq were performed on 1256 samples from the GCEP study cohort (with a minimum follow up period of 5 years), while additional samples (not from GCEP1000) were profiled using WES, RNA-seq, scRNA-seq, and spatial transcriptomics. In the GCEP1000 cohort, the majority of subjects (88.9%; 573/644) were Hp-positive by serology indicative of previous Hp exposure, and upon GCEP enrollment subjects with evidence of active Hp infection were treated for Hp eradication (114/644, diagnosed by histology). IM cases exhibiting high-HP levels by panel sequencing corresponded to samples collected i) prior to Hp eradication, ii) samples where eradication was performed but not completely successful, and iii) cases of re-infection.

To enable intra-patient (i.e., within-patient) comparisons, we profiled samples from multiple stomach sites (antrum: n = 642; body: n = 274; cardia: n = 265). A subset of samples was matched from the same subjects across time, enabling longitudinal comparisons from i) subjects who developed dysplasia during their course of observation (n = 64), ii) had concurrent dysplasia (n = 93), or iii) exhibited dysplasia regression (n = 98)) (Figure 1A). Selected GCEP1000 samples with appreciable median VAFs were analyzed by whole-genome sequencing (WGS, n = 5) or WES (n = 52) to assess mutational counts, signatures, and sCNAs. At the transcriptomic level, we performed bulk RNA-sequencing on 183 GCEP1000 samples, including normal (n = 46) and IMs (n = 137) from multiple sites (antrum: n = 55; body: n = 66; cardia: n = 62).

To complement the GCEP1000 data, we further generated a) WES and RNA-seq data of 28 cases of concurrent normal, dysplasia and early GC from South Korea, b) scRNA-seq from 18 patients with antral gastric biopsies and six patients with body/cardia gastric biopsies to survey tissue ecologies, and c) Nanostring Digital Spatial Profiling (DSP) spatial profiles of eight patients whose antral sections contained histologically normal, IM, GC, lymphoid aggregates, and stromal regions, representing 480 ROIs and 76 CD45-segmented AOIs (Table S1).

#### Selection of genomic targets

Candidate genes for GCEP1000 targeted sequencing were selected from a literature review of candidate genes that were (1) significantly mutated or exhibiting copy number alterations in gastrointestinal adenocarcinoma, (2) commonly mutated in gastrointestinal adenocarcinoma, and (3) significantly mutated or copy number altered in pre-malignant, inflamed or normal tissues. A total of 277 human genes were selected. We included 6 Hp genes and ∼5000 SNPs distributed across the genome to identify Hp infection and sCNAs. Agilent SureSelect E-array software was used to design unique RNA baits for the gene panel. Biotinylated RNA baits were synthesized by Agilent for use with the SureSelect Target Enrichment system (Agilent, USA).

#### GCEP1000 bulk DNA and RNA extraction and library preparation

Genomic DNA from frozen tissues and blood samples were extracted using the Wizard Genomic DNA Purification Kit (Promega, Madison, Wisconsin, USA) or QIAamp DNA Mini Kit (Qiagen, Hilden, Germany) according to manufacturer protocols. For samples selected for RNA-seq, genomic DNA and total RNA were extracted simultaneously from tissues using the AllPrep DNA/RNA Micro Kit (Qiagen) according to the manufacturer’s protocol.

DNA samples were quantified using Qubit broad range assays (Thermo: Q32853) and qualified using Genomic DNA ScreenTapes on a Tapestation (Agilent, 5067–5365). The target enrichment platform was Agilent SureSelect XT HS2 DNA System with Pre-Capture Pooling (Agilent: G9985A, G9985B, G9985C, G9985D) with a customized tier 2 design. Briefly, 100 ng of DNA from each sample was enzymatically fragmented (Agilent: 5191-4080) before end-repair, ligation of adapters and pre-capture amplification using unique dual indexing primer pairs. The yield and size distribution of each sample was checked using D1000 ScreenTapes (Agilent: 5067–5582). 16 samples were pooled in equal amounts to 1.5 μg per hybridization with the custom panel following manufacturer instructions. The hybridized DNA samples were captured using streptavidin-coated beads before amplification. The yield and size distribution of the captured samples were analyzed on High Sensitivity ScreenTapes (Agilent: 5067–5584). The libraries were sequenced on Illumina Novaseq 6000 equipment (PE150 bp), according to manufacturer protocols.

Whole genome sequencing libraries were constructed using the New England Biolabs NEBNext Ultra II DNA Library Prep Kit. The genomic DNA was randomly sheared into short fragments, and the obtained fragments were end-repaired, A-tailed, and further ligated with Illumina adapters. The fragments with adapters were PCR amplified, size selected, and purified. The libraries were checked with Qubit and real-time PCR for quantification and on an Agilent bioanalyzer for size distribution detection. Quantified libraries were pooled and sequenced on the Illumina Novaseq 6000 (PE150 bp) according to manufacturer’s protocols.

10 ng of total RNA was used to create RNA-seq libraries using the SMART-Seq Stranded Kit (Takara Bio USA, Mountain View, California, USA) according to the manufacturer protocols. Library fragment size was determined using the High Sensitivity Kit on the Agilent Bioanalyzer (Agilent Technologies). The libraries were sequenced on an Illumina Novaseq 6000 (PE150 bp) according to manufacturer protocols.

#### GCEP1000 DNA sequencing analysis

Targeted sequencing reads were aligned to the human reference genome hg37 using BWA MEM.^69^ Duplicates were removed with Agilent’s AGeNT tool using molecular barcode information. Aligned BAM files were further processed according to GATK^70^ Best Practices guidelines. We used Mutect2 due to its higher sensitivity in detecting low frequency variants from high depth sequencing data.^71^^,^^72^^,^^73^ The Mutect2 options “--force-active true --pruning-lod-threshold −4 --max-reads-per-alignment-start 0” were further used to improve sensitivity at the expense of runtime. To balance specificity, standard Mutect2 somatic variant filters were applied to remove background germline variations and sequencing artifacts using the Genome Aggregation Database (gnomAD) germline resource and a panel of normals (PoN) consisting of 726 germline samples profiled on the same panel. Additional filters included checking for cross-sample contamination (GATK4’s CalculateContamination) and filtering for possible read-orientation sequencing artifacts (GATK4’s CollectF1R2Counts and LearnReadOrientationModel). Finally, somatic variants with at least 5 variant supporting reads were included as the final dataset of high-confidence calls.

We also compared the Mutect2 mutation calls with two other mutation callers - Varscan2^74^ and Strelka2.^75^ The majority of driver gene mutations identified with Mutect2 were also identified using either Varscan2 and/or Strelka2 (1406/2173; 64.7%). Unsurprisingly, mutations with higher allele frequencies (>1%) were more likely to be validated by Varscan2/Strelka2 (995/1179; 84.4%). Of the 994 low frequency mutations (VAF<1%), 282 are predicted to be oncogenic/likely oncogenic alterations (OncoKB) and 64 coincide with reported cancer hotspot sites (e.g., *KRAS* G13D, *FBXW7* R465H and *ERBB2* S310F mutations), suggesting these are likely to be true and functionally impactful. Examination of mutational signatures in mutations with high or low VAFs confirmed a similar mutation profile dominated by SBS1 (78 vs. 79%) and SBS3+8 (10 vs. 9%) (Figure S1E and S1F).

The functional effects of variants were annotated using Funcotator.^76^ Genes under positive selection were identified using dNdScv,^22^ by analyzing the ratio of nonsynonymous to synonymous mutations. For comparison, we also included driver gene analysis using two other tools MutSigCV^77^ and OncoDriveFML.^78^ Notably, 22/26 driver genes identified with dNdSCV were also identified with MutSigCV or OncodriveFML, suggesting a broad concordance between different driver gene algorithms (Table S3). We also conducted a separate dN/dS driver mutation analysis categorized by their VAFs. When restricted to only somatic mutations with VAF>1%, 23/26 predicted drivers remained significant (exceptions being *KLF5*, *ARID1B*, and *MAP3K1*). When restricted to only mutations with VAF<1%, 9 driver genes remained significant (*ARID1A* (number of protein-altering mutations, n = 97), *SOX9* (n = 67), *ARID2* (n = 60), *CTNNB1* (n = 44), *KRAS* (n = 15), *PIGR* (n = 36), *FBXW7* (n = 36), *ERBB3* (n = 36), *CCND1* (n = 12)), consistent with these lower frequency mutation exhibiting functional relevance. Protein altering mutations in these 9 genes were rarely observed in blood samples (56/682; 8.2% blood vs. 537/1217; 44.1% in gastric biopsies, Fisher-test p value<2.2 × 10^−16^) sequenced and processed in the same way as the gastric biopsies, further indicating that the low frequency mutations are genuine driver mutations arising from small clonal populations. We determined clone sizes as twice the mutation VAF79 and estimated the fractional size of a gastric tissue covered by mutant drivers from the total summed size of driver clones in each biopsy (capped at 1.0).^79^

sCNAs were analyzed using two approaches, the GATK ACNV workflow and ASCAT.^80^ Raw copy ratio and allelic copy ratios at targeted regions were collected for both IM and matched blood samples. For GATK, amplified or lost segments were called using CallCopyRatioSegments with default parameters and according to GATK best practices. Allele-specific copy number profiles were separately generated using ASCAT. For ASCAT, Log R ratio (LogR) and B-allele frequency (BAF) plots were manually inspected to select for highly confident sCNAs. GCEP1000 samples (n = 52) with detectable sCNAs and sufficient DNA material were further profiled using WES to validate identified sCNAs (average WES coverage 180X). We validated 88% of the 7q and 8q amplifications by WES (7q: 19/19; 8q: 9/13) (Figure S2C). 8p and 11p deletions were less supported possibly due to lower WES coverage (8p:2/18; 11p:3/6).

For WGS data, sequencing reads were aligned using BWA MEM and processed using GATK, including duplicate removal using MarkDuplicate, local read realignment and base quality score recalibration. Variant calling was performed using standard Mutect2 commands comparing BAM files for IM samples compared to matched blood samples. Mutational signatures were fitted using the signature.tools.lib^81^ R package with stomach-specific substitution as reference.

#### Bulk RNA-sequencing analysis

Sequencing reads were aligned to human reference sequence hg37 using Hisat2,^82^ and gene expression was quantified using Stringtie.^83^ Differential gene expression and gene set enrichment analysis was performed using DESeq2^84^ and fgsea (https://github.com/ctlab/fgsea) respectively. Stemness features were estimated using the TCGAanalyze_Stemness^85^ function.

To quantify bacterial microbiomes, we applied PathSeq^44^ to first remove all reads aligned to the human genome, followed by realignment of the remaining reads (with a minimum clipped read-length of 100 bp) to the NCBI database of bacterial reference genomes. Reads that were unambiguously aligned to specific bacterial genera were retained for analysis and the abundance of each bacteria genus normalized to the total number of sequencing reads aligned to the human genome. To estimate bacterial diversity, we used PathSeq normalized (%) scores which are normalized to microbial genome sizes and the overall bacterial scores. All other comparisons used the absolute numbers of unambiguous reads mapping to genus X/reads mapping to human. Oral bacteria was defined by querying their prevalence in the healthy oral cavity and stomach using the mBodyMap^86^ database. We focused our analysis on the 30 most abundant bacterial genera which are likely to be more accurately quantified. We also confirmed a high concordance between *Helicobacter* abundance in our RNA-seq data with histological results (Figure S7A).

#### Whole exome sequencing

DNA samples from patients with concurrent adjacent normal, dysplasia and GC were quantified using Qubit brand range assays (Thermo: Q32853) and qualified using Genomic DNA ScreenTapes on a Tapestation (Agilent, 5067–5365). The target enrichment platform used was Agilent SureSelect XT HS2 DNA System with Pre-Capture Pooling (Agilent: G9985A, G9985B) with Agilent SureSelect Human All Exon V6 (5190–8873). Briefly, 100 ng of DNA from each sample was enzymatically fragmented (Agilent: 5191-4080) before end-repair, ligation of adapters and pre-capture amplification with unique dual indexing primer pairs. The yield and size distribution of each sample was checked on D1000 ScreenTapes (Agilent: 5067–5582). 8 samples were pooled in equal amounts to 1.5 μg per hybridization with the SureSelect Human All Exon V6 probes following manufacturer instructions. Hybridization temperature was set at 62.5°C as recommended. The hybridized DNA samples were captured using streptavidin-coated beads before amplification. The yield and size distribution of the captured samples were analyzed on High Sensitivity ScreenTapes (Agilent: 5067–5584). The libraries were also checked with Qubit and real-time PCR for quantification. The quantified libraries were pooled and sequenced on the illumina Novaseq 6000 (PE150 bp), according to manufacturer’s protocol.

Exome sequencing reads were aligned to the reference human genome hg37 using BWA MEM. Preprocessing steps including duplicate marking, local read realignment and base quality score recalibration were performed using Picard and Genome Analysis Toolkit (GATK) to generate analysis-ready BAM files. Mutect2 was used in paired mode to generate a list of somatic SNVs and indels in the 277 genes used in the GCEP1000 panel. GCs were classified as EBV, MSI, CIN or GS using previously proposed classification systems.^15^ EBV-positive tumors were identified by evaluating the number of reads mapping to the NC_007605 EBV genome. MSI status was assessed using MSIsensor2,^87^ a tool for detecting microsatellite instability from sequencing data. sCNAs in the exome data were identified using the GATK ACNV method. Significant somatic copy number alterations in GC samples were identified using GISTIC2.^88^ Hierarchical clustering was performed on tumors using copy number profiles from significant copy number altered regions from GISTIC2. Clusters with higher sCNA burden were considered as CIN-positive and the remaining samples were considered as GS tumors.

#### Cell lines

GES-1 cells were a gift from Dr Alfred Cheng, Chinese University of Hong Kong. HFE-145 cells were obtained from Dr Hassan Ashktorab, Howard University. SNU-484 cells were from the Korean Cell Line Bank. All lines were negative for mycoplasma contamination as assessed by the MycoAlert Mycoplasma Detection Kit (Lonza).

#### Generation of C-terminal deleted mutant cells

We used the Multiplex CRISPR/Cas9 Assembly System Kit to generate *SOX9* C-terminal deleted mutant cells. The gRNA sequences were subcloned into insert plasmid px330S-2 and vector plasmid pX330A-Cas9-2A-GFP-1x2 plasmid expressing Cas9 nuclease. The expression cassette from plasmid px330S-2 with the gRNA was then subcloned into pX330A-Cas9-2A-GFP-1x2. Sequences were: sgRNA 1 (5′ end of Exon 3), sense 5′-ATTGTCCACAGGGCAATCCC-3′; antisense 5′-GGGATTGCCCTGTGGACAAT-3′, sgRNA 2 (3′ end of Exon 3), sense 5′-ACACAGCTCACTCGACCTTG-3’; antisense 5′-CAAGGTCGAGTGAGCTGTGT-3′. All the gRNAs had no potential off-target sites. Plasmids at 500 ng/μL concentration were used for cell transfection using Lipofectamine 3000 transfection kit (Invitrogen). GFP-positive cells were sorted by a BD FACS Aria (BD Bioscience). Cell clones were cultured for 2–3 weeks, and the DNA was isolated for genotype verification. PCR amplification of the DNA sample was performed using KOD FX (Toyobo). PCR products were purified with PCR GEL/PCR Purification Mini Kit (Favorgen) and sent for DNA Sanger sequencing (1st BASE). Sequences of primers for genotyping: forward 5′-AAGTAGCAATTAGGTCTTCCGGACCC-3’; reverse 5′-ATCTCTCTCCACGCTTGCTCTG-3’.

#### RNA isolation and qRT-PCR

Total RNA from samples was isolated with TRIzol reagent (Invitrogen) and reverse transcribed with ReverTra Ace qPCR RT Master Mix with gDNA Remover (Toyobo) according to the manufacturer’s instructions. qRT-PCR was performed using PowerUp SYBR Green Master Mix (Life Technologies) with the following primers: *SOX9*, forward 5′-GGCAAGCTCTGGAGACTTCTG -3′, reverse 5′-CCCGTTCTTCACCGACTTCC-3′, *β-Actin*, forward 5′- CATGTACGTTGCTATCCAGGC-3′, reserve 5′- CTCCTTAATGTCACGCACGAT-3’.

#### Cell proliferation assay

500 - 1000 cells were seeded in 96-well plates for cell proliferation assays and measured using a Cell Counting Kit-8 (Dojindo) for 4 to 5 days 10 μL Cell Counting Kit-8 solution was added to each well and incubated for 1–2 h in a humidified incubator. Absorbance readings were measured using a Tecan Infinite M200 Microplate Reader. The readings for each day were normalized to Day 0 to generate growth curves for further comparisons.

#### Bulk RNA-Sequencing and analysis

Bulk RNA-seq and analysis was performed following a similar methodology to that employed for GCEP1000 samples.

#### Single-cell RNA-sequencing

Patients with IM undergoing endoscopic biopsies at the National University Hospital or Tan Tock Seng Hospital, Singapore were enrolled after written informed consent. Tissues were collected in MACS tissue storage solution (Miltenyi Biotec) immediately after biopsy and stored on ice. Tissue processing was performed as previously reported.^30^ Samples were processed using enzymatic and mechanical dissociation with a human tumor dissociation kit and the Gentle MACS Octodissociator (Miltenyi Biotec) following the manufacturer’s “37_h_TDK_2” program. The dissociated cells were passed through a MACS smartstrainer (70 μm) and incubated with RBC lysis buffer for 5 min followed by PBS neutralization. All centrifugation steps were carried out at 300×g for 7 min. Dissociated cells were washed twice in PBS +1% bovine serum albumin (BSA). Live-cell counts were obtained by manual cell counting using 1:1 trypan blue dilution. Cells were concentrated to 800–1,200 live cells/μL and processed for single-cell analysis.

Samples from each patient were processed in a single batch for library preparation. The Chromium Single-Cell 3′ Library and Gel Bead Kit (10× Genomics) was used according to the manufacturer’s protocols. Each cell suspension sample was loaded into a chromium next GEM chip G for a target cell output of 10,000 cells. Gel bead-based emulsions (GEMs) were generated by combining cells, barcoded single-cell 3′ Gel Beads and partitioning oil. Post GEM-RT samples were cleaned up by Dynabeads, and 10× barcoded full-length cDNAs generated from GEMs were amplified by PCR. Amplified cDNAs were cleaned up using SPRIselect beads (Beckman Coulter) and analyzed by Agilent Bioanalyzer High Sensitivity DNA chips (Agilent Technologies) to calculate the total cDNA yield. Twenty-five percent of the cleaned up amplified cDNA samples were fragmented followed by adapter ligation and index PCR for a total of 14 cycles. Enriched libraries were enzymatically digested, size selected, and adapter ligated for sequencing. Quantified libraries were sequenced on a Hiseq4000 (Illumina).

Raw fastq sequencing data for each sample was processed using the Cell Ranger software (https://support.10xgenomics.com/single-cell-gene-expression/software/downloads/latest#cellrangertab) onto the human hg37 reference genome to generate a gene expression count matrix. Subsequently, Seurat^89^ was utilized to perform basic quality control (QC) filtering. Genes shared by fewer than 3 cells and cells with fewer than 500 or more than 7000 genes were filtered out using the Seuratsubset function. Cells with mitochondrial RNA percentage (MT%) higher 50% were also filtered. DoubletFinder^90^ was employed for each sample to remove potential doublet cells. Processed samples were integrated using the Seuratmerge function, normalized using SeuratSCTransform, and scaled and analyzed by principal component analysis (PCA). The data were visualized using the Uniform Manifold Approximation and Projection (UMAP) method, and cells were clustered using the Seurat shared nearest neighbor (SNN) algorithm with the Leiden-graph approach.

GC scRNA-seq profiles from our previous study^30^ were processed separately, following the same workflow as described in the previous section. sCNA analysis was performed on each sample using CopyKAT^91^ by setting a pool of matched normal cells as reference normal cells. The predicted aneuploid cells were then sub-clustered into sub-groups in a heatmap based on their Euclidian distances on the sCNA matrix, and a phylogenetic neighbor joining (NJ) tree was set up using the R package phangorn.^92^ The NJ tree was re-rooted using a defined diploid cell. Based on the clustering heatmap and the NJ tree, the predicted aneuploid cells with lower levels of sCNA burden and closer to the root in the NJ tree were defined as “early stage tumor cells”. The raw count data of the IM cells and early stage tumor cells were integrated using the Seuratmerge function. The integrated data were then processed following the same workflow as described earlier. Monocle3^93^ was used to perform trajectory analysis on this data with default parameters. The embeddings of Monocle3 cell dataset (CDS) object were replaced using the Seurat UMAP embeddings for consistency. The root cells of the CDS object were manually selected in intestinal stem cells.

#### Digital spatial profiling

Formalin fixed, paraffin-embedded (FFPE) blocks were cut into five micrometer sections and mounted on BOND Plus slides (Leica Biosystems, Wetzlar, Germany). Hematoxylin and eosin (H&E) staining was performed on one slide scanned at 10X magnification with Metafer 4 software (MetaSystems, Altlussheim, Germany). For ROI selection, a certified pathologist (SS) selected tumor areas, normal glandular tissue, IM positive areas, stroma rich areas and lymphoid aggregates on the digitally scanned H&E slides. A consecutive slide was then processed according to GeoMx Human Whole Transcriptome Atlas (NanoString, Seattle, WA, United States) methodology. The selected areas (on H&E) were used to annotate the GeoMx slide with the aid of SYTO13 and PanCK (for tumor rich areas, normal glandular tissue and IM positive areas), Smooth Muscle Actin (for stroma) and CD45 (for lymphoid aggregates) markers. Subsets of ROIs were segmented to generate custom AOIs (CD45^+^ and CD45^−^regions). 22 to 95 ROIs/AOIs were selected for each slide. Libraries were constructed using Seqcode reagents (NanoString, Seattle, WA, United States) and sequenced on the Illumina platform.

FASTQ files for DSP profiles were processed to generate count matrices as previously described.^94^ Briefly, deduplicated sequencing counts were calculated based on UMI and molecular target tag sequences. Single-probe genes were reported as the deduplicated count value. Count data were processed and normalized using the GeoMxTools R package. AOIs/ROIs with fewer than 1000 raw reads or the percentage of aligned reads <75% or a sequencing saturation <50% were filtered out of the analysis. The limit of quantitation was estimated as 2 geometric standard deviations of the negative control probes above the geometric mean of the negative control probes. AOIs/ROIs that had only a small percentage (<5%) of panel genes detected above the quantitation limit were removed, and the genes with a low detection rate (<10%) among the remaining AOIs/ROIs were filtered out. The datasets were normalized using upper quartile (Q3) normalization. To estimate cell abundances in each AOI/ROI, the SpatialDecon^95^ algorithm was employed using safeTME as a cell-profile matrix.

To distinguish between intestinal stem-cell dominant IM and enterocyte dominant IM, we used Seurat FindMarker to select the top 500 markers (for IM-enterocyte and IM-stem cells each) from our scRNA-seq data. GSEA was performed using these markers for each IM region, which were normalized to the average gene expression in histologically normal samples to annotate IM regions as IM-enterocyte dominant or IM-stem cell dominant. Unsupervised clustering was performed on the 1000 selected markers (500 IM-Enterocyte and 500 IM-stem cell) using Euclidean distance and Ward.D2 clustering.

#### Clinical model

Logistic regression analysis was used to predict the risk of dysplasia. The clinical risk stratification model was based on four established clinical risk factors - age, pepsinogen index, OLGIM score, and smoking status. Molecular test results such as mutation counts, clone sizes, and sCNAs, were further incorporated into the clinical model to test for its capability to provide additional information on risk prediction beyond present clinical and histological information. ROC curves were used to present the performance of risk factors, with AUC values as the performance indicator. All statistical analysis was performed using IBM SPSS Statistics 28 (IBM Corp., Armonk, NY, USA). A p value of less than 0.05 was considered statistically significant.

We also investigated the possibility of using microbial data as an additional prognosis marker, by extending the genomic-clinical analysis to bacterial levels inferred using targeted DNA sequencing (Figures S8A and S8B). While our initial results might imply that gut bacterial markers do not contribute to models of predicting IM progression to GC, this finding should be treated with caution due to the lower sensitivity of detecting bacterial reads from the targeted sequencing compared to RNA-seq, resulting in a zero-inflated dataset that may not be amenable for logistic regression analysis.

### Quantification and statistical analysis

In addition to the algorithms described above, all other basic statistical analyses were performed in the R statistical environment (v4.2.0). All statistical tests performed in this study were two-sided and p values <0.05 were considered statistically significant, unless otherwise stated. Differential gene expression analysis was carried out using DESeq2 on raw count data generated using Stringtie or obtained from TCGA. For scRNA-seq, differential analysis was performed using Seurat’s FindMarker function. P-values for DESeq2 and FindMarker were adjusted for multiple comparisons using their respective default settings. Differential abundance of microbiomes was analyzed using lefser with default parameters. Statistical analysis for cell line work was performed using GraphPad Prism Version 10.0 software package (GraphPad Software, San Diego, CA). Mann-Whitney non-parametric two-tailed unpaired t-tests were used for two-group gene expression comparisons. two-way ANOVA with Geisser-Greenhouse correction and Tukey’s multiple comparison tests were used for the cell proliferation assay. We have provided the statistical tests used (Fisher-test, Wilcoxon-test and Spearman correlation) in the figures or figure legends.

## Data Availability

Raw sequencing data, including GCEP1000 panel targeted sequencing, bulk RNA sequencing, whole genome sequencing, single-cell RNA sequencing and digital spatial transcriptomics has been deposited at the European Genome-phenome Archive (EGA) under accession number EGA: EGAS00001007067. Any additional information required to reanalyse the data reported in this paper is available from the lead contact upon request.

## Supplemental information

Document S1. Figures S1—S8

Table S1. Clinicopathological features of GCEP1000 cohort (DNA-seq and RNA-seq), single-cell, spatial transcriptomic and whole exome sequencing samples, related to Figure 1

Table S2. Driver genes identified using dNdScv, MutSigCV and OncodriveFML, related to Figure 1

Table S3. Driver mutation rate in normal, IM and GC samples across various cohorts including GCEP1000 validation cohort, related to Figure 1

Table S4. Univariate and multivariate analysis of the association between driver alterations and dysplasia, related to Figure 7 Factors with p < 0.15 in univariable analysis were assessed in the multivariate analysis.

Document S2. Article plus supplemental information
