# Supplementary material for: Spatiotemporal genomic profiling of intestinal metaplasia reveals clonal dynamics of gastric cancer progression
Source: Cancer Cell. 2023 Dec 11;41(12):2019–2037.e8. doi: 10.1016/j.ccell.2023.10.004 (PMC10729843; doi:10.1016/j.ccell.2023.10.004)
Supplement: Document S1. Figures S1—S8 [file mmc1.pdf]

Cancer Cell, Volume *41*

Supplemental information

Spatiotemporal genomic profiling of intestinal metaplasia reveals clonal dynamics of gastric cancer progression

Kie Kyon Huang, Haoran Ma, Roxanne Hui Heng Chong, Tomoyuki Uchihara, Benedict Shi Xiang Lian, Feng Zhu, Taotao Sheng, Supriya Srivastava, Su Ting Tay, Raghav Sundar, Angie Lay Keng Tan, Xuewen Ong, Minghui Lee, Shamaine Wei Ting Ho, Tom Lesluyes, Hassan Ashktorab, Duane Smoot, Peter Van Loo, Joy Shijia Chua, Kalpana Ramnarayanan, Louis Ho Shing Lau, Takuji Gotoda, Hyun Soo Kim, Tiing Leong Ang, Christopher Khor, Jonathan Wei Jie Lee, Stephen Kin Kwok Tsao, Wei Lyn Yang, Ming Teh, Hyunsoo Chung, Jimmy Bok Yan So, Khay Guan Yeoh, Patrick Tan, and Singapore Gastric Cancer Consortium

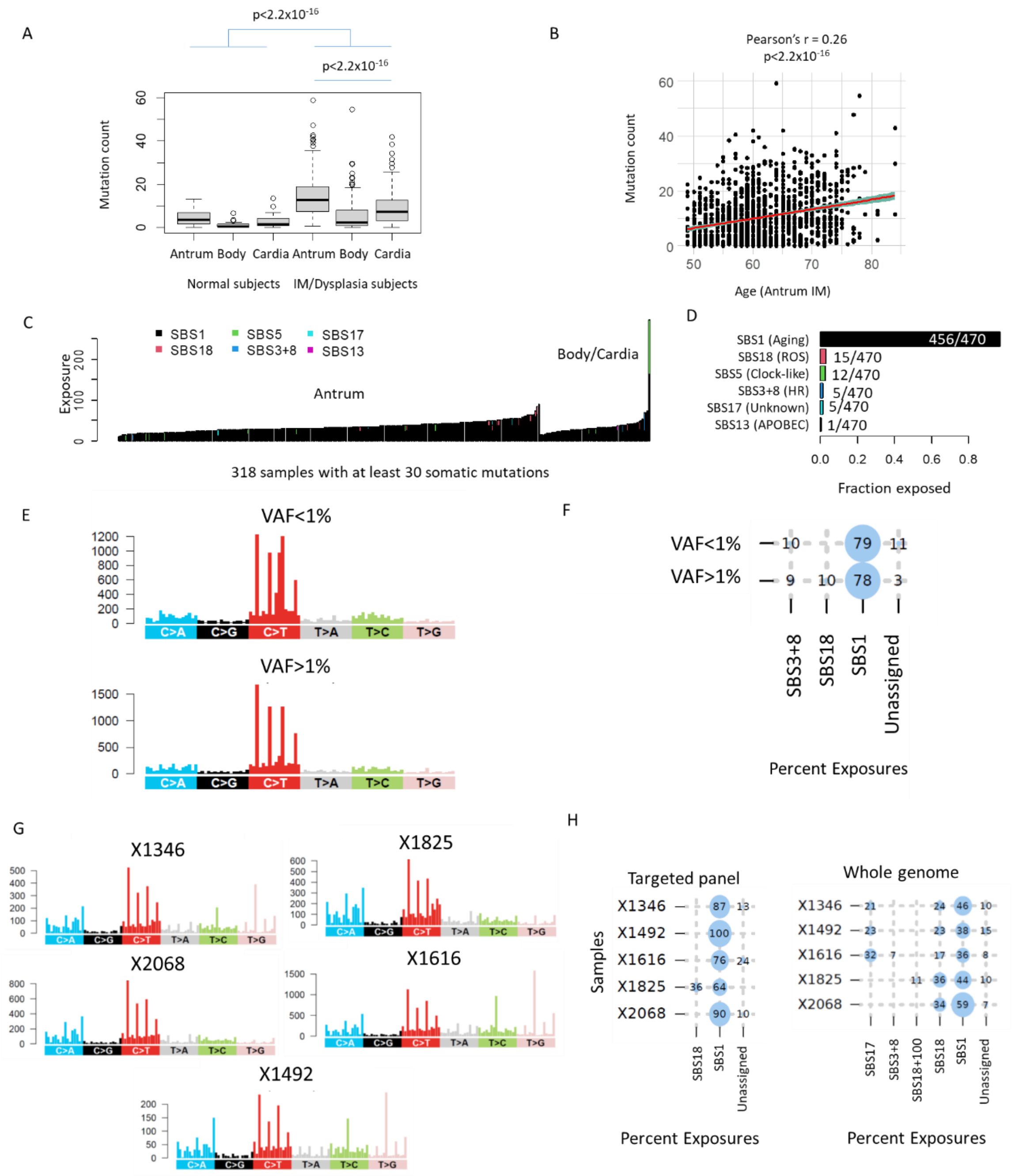

Figure S1. Genomic landscape of IM and normal samples. Related to Figure 1. (A) Mutation rates of IM and normal samples at different sites. (B) Correlation between antral IM mutation counts and age. (C) Mutation signatures detected from IM samples. (D) Fraction of IM samples with detectable mutational signatures. (E) Signatures are similar between mutations of high (>1%) and low (<1%) VAF. (F) Proportional exposures to mutation signatures in mutations of high (>1%) and low (<1%) VAF. (G) Mutation spectrum from 5 antral IMs profiled using WGS. (H) Proportional exposures to mutation signatures in 5 antral IMs from the GCEP1000 targeted panel and WGS.

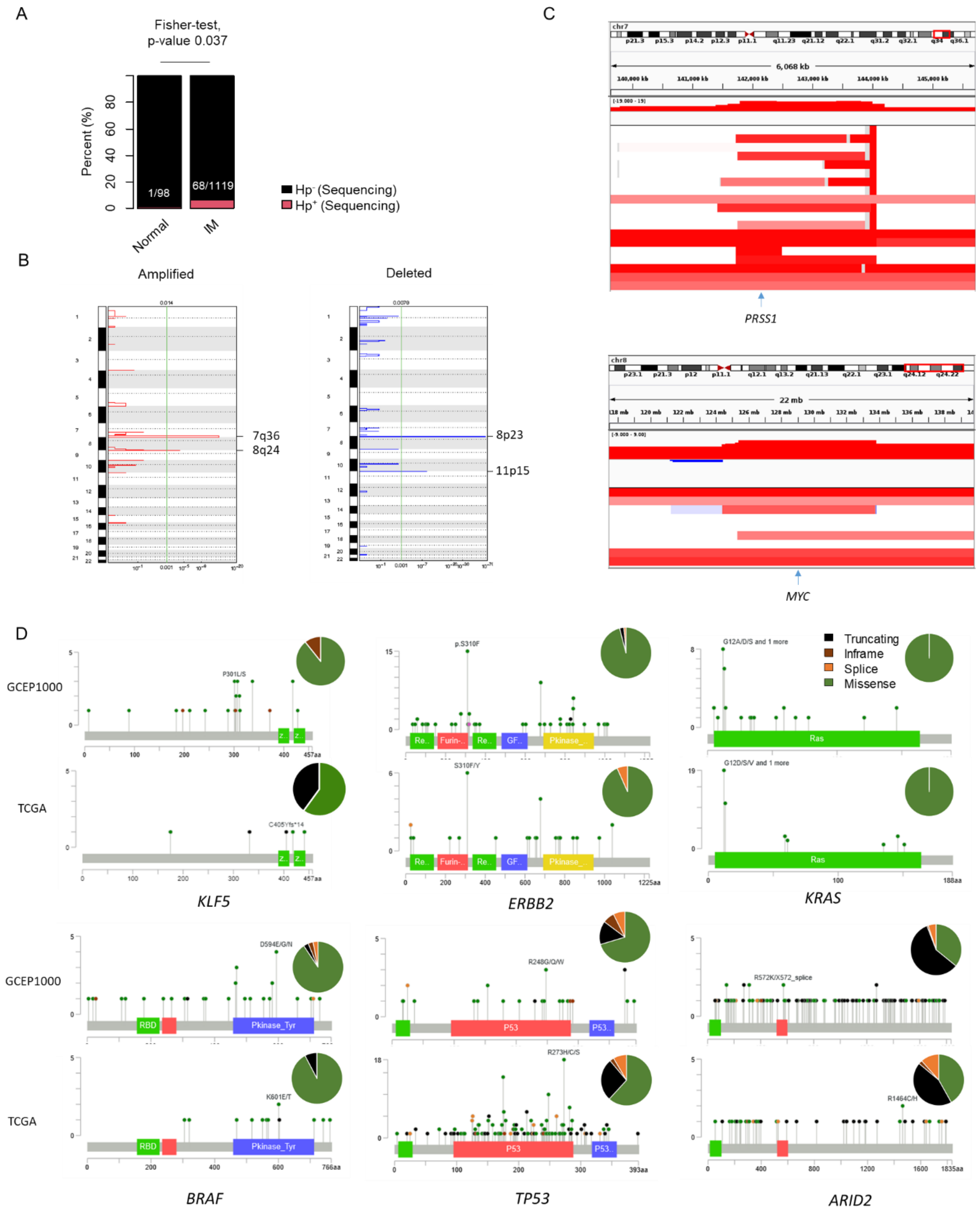

Figure S2. Driver alterations in IM. Related to Figure 1. (A) Hp detection by targeted DNA-seq in normal and IM samples. (B) GISTIC amplification and deletion sCNA regions identified by GATK. (C) Amplified regions in chromosome 7q (left) and 8q (right) validated with WES. Gene locations of *MYC* and *PRSS1* are indicated by arrows. (D) Lollipop plots highlighting genomic alterations in selected genes in pre-malignant IM (GCEP) and GC (TCGA)

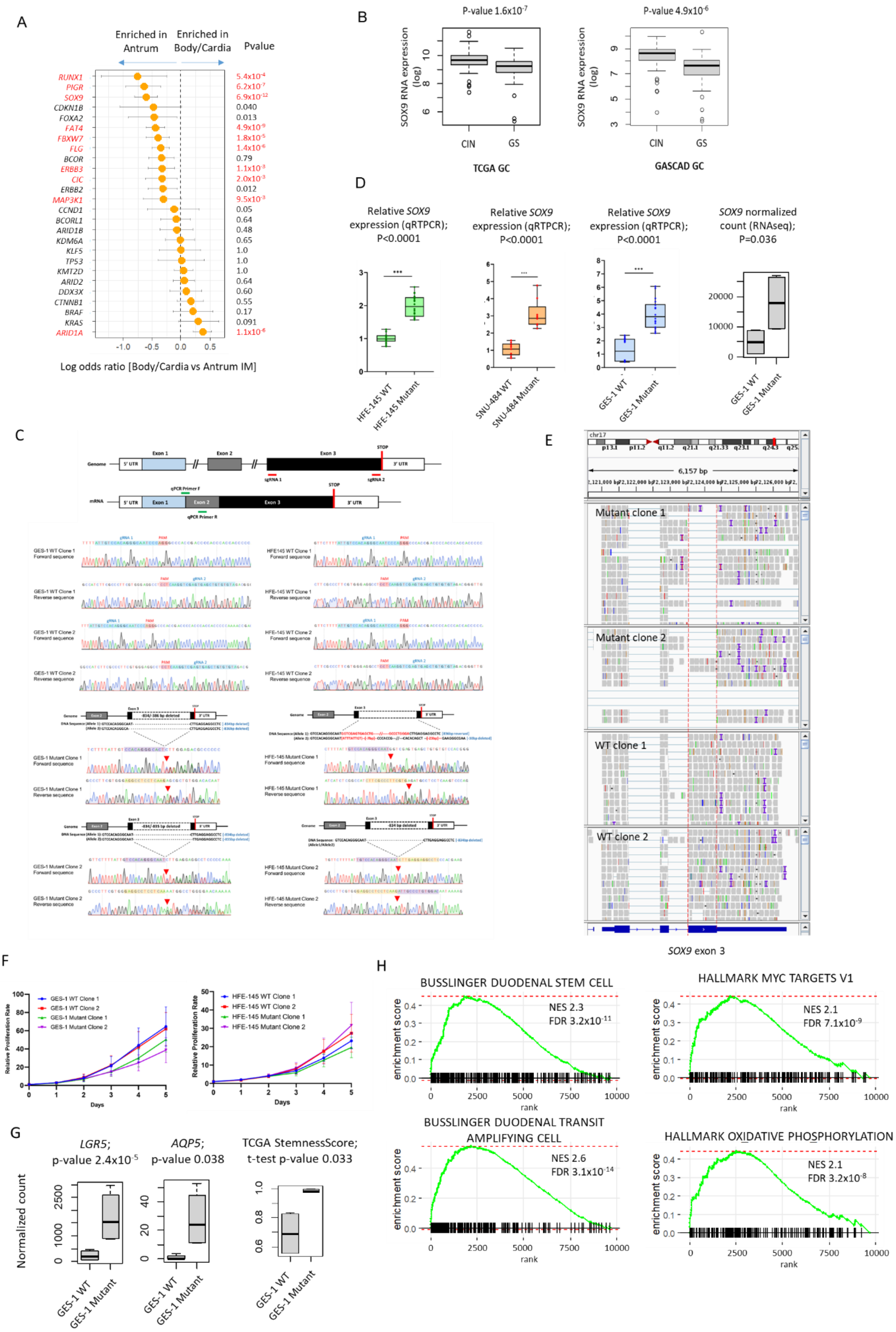
A
Enriched in Antrum
Enriched in Body/Cardia
Pvalue
RUNX1 5.4x10-4
PIGR 6.2x10-7
SOX9 6.9x10-12
CDKN1B 0.040
FOXA2 0.013
FAT4 4.9x10-9
FBXW7 1.8x10-5
FLG 1.4x10-6
BCOR 0.79
ERBB3 1.1x10-3
CIC 2.0x10-3
ERBB2 0.012
MAP3K1 9.5x10-3
CCND1 0.05
BCORL1 0.64
ARID1B 0.48
KDM6A 0.65
KLF5 1.0
TP53 1.0
KMT2D 1.0
ARID2 0.64
DDX3X 0.60
CTNNB1 0.55
BRAF 0.17
KRAS 0.091
ARID1A 1.1x10-6
Log odds ratio [Body/Cardia vs Antrum IM]
B
P-value 1.6x10-7
P-value 4.9x10-6
SOX9 RNA expression (log)
CIN
GS
TCGA GC
GASCAD GC
C
D
Relative SOX9 expression (qRTPCR); P<0.0001
Relative SOX9 expression (qRTPCR); P<0.0001
Relative SOX9 expression (qRTPCR); P<0.0001
SOX9 normalized count (RNAseq); P=0.036
HFE-145 WT
HFE-145 Mutant
SNU-484 WT
SNU-484 Mutant
GES-1 WT
GES-1 Mutant
E
Mutant clone 1
Mutant clone 2
WT clone 1
WT clone 2
SOX9 exon 3
F
GES-1 WT Clone 1
GES-1 WT Clone 2
GES-1 Mutant Clone 1
GES-1 Mutant Clone 2
HFE-145 WT Clone 1
HFE-145 WT Clone 2
HFE-145 Mutant Clone 1
HFE-145 Mutant Clone 2
Relative Proliferation Rate
Days
G
LGR5; p-value 2.4x10-5
AQP5; p-value 0.038
TCGA StemnessScore; t-test p-value 0.033
Normalized count
H
BUSSLINGER DUODENAL STEM CELL
NES 2.3
FDR 3.2x10-11
HALLMARK MYC TARGETS V1
NES 2.1
FDR 7.1x10-9
BUSSLINGER DUODENAL TRANSIT AMPLIFYING CELL
NES 2.6
FDR 3.1x10-14
HALLMARK OXIDATIVE PHOSPHORYLATION
NES 2.1
FDR 3.2x10-8
enrichment score
rank

Figure S3. *SOX9* mutations in IM. Related to Figure 1. (A) IM driver mutation rates by stomach sites. *SOX9* mutations are enriched in antral IM. P-values were estimated using Fisher tests. (B) Expression in CIN vs genome-stable (GS) TCGA (left) and GASCAD (right) GC samples. P-values were estimated using DESeq2. (C) *SOX9* regions targeted for functional analysis (red arrows) with Sanger validation (D) Loss of the *SOX9* C-terminus results in higher *SOX9* gene expression. P-values were estimated using Mann-Whitney tests for RT-PCR and DESeq2 for RNAseq. (E) IGV view of *SOX9* transcripts in WT and mutant clones. Mutant clones largely express *SOX9* with truncated C-termini (exon 3). (F) *SOX9* C-terminal loss does not affect cell proliferation. (G) Normalized counts of *LGR5* and *AQP5* (left) and TCGA stemnessScore (right) in wildtype GES-1 (WT) and mutant *SOX9* clones. P-values were estimated using DeSEQ2 (*LGR5* and *AQP5* expression) and Welch t-tests for stemness scores. (H) GSEA plots showing enrichment of intestinal stem cell signatures, *MYC* and oxidative phosphorylation pathway genes in *SOX9* mutant clones.

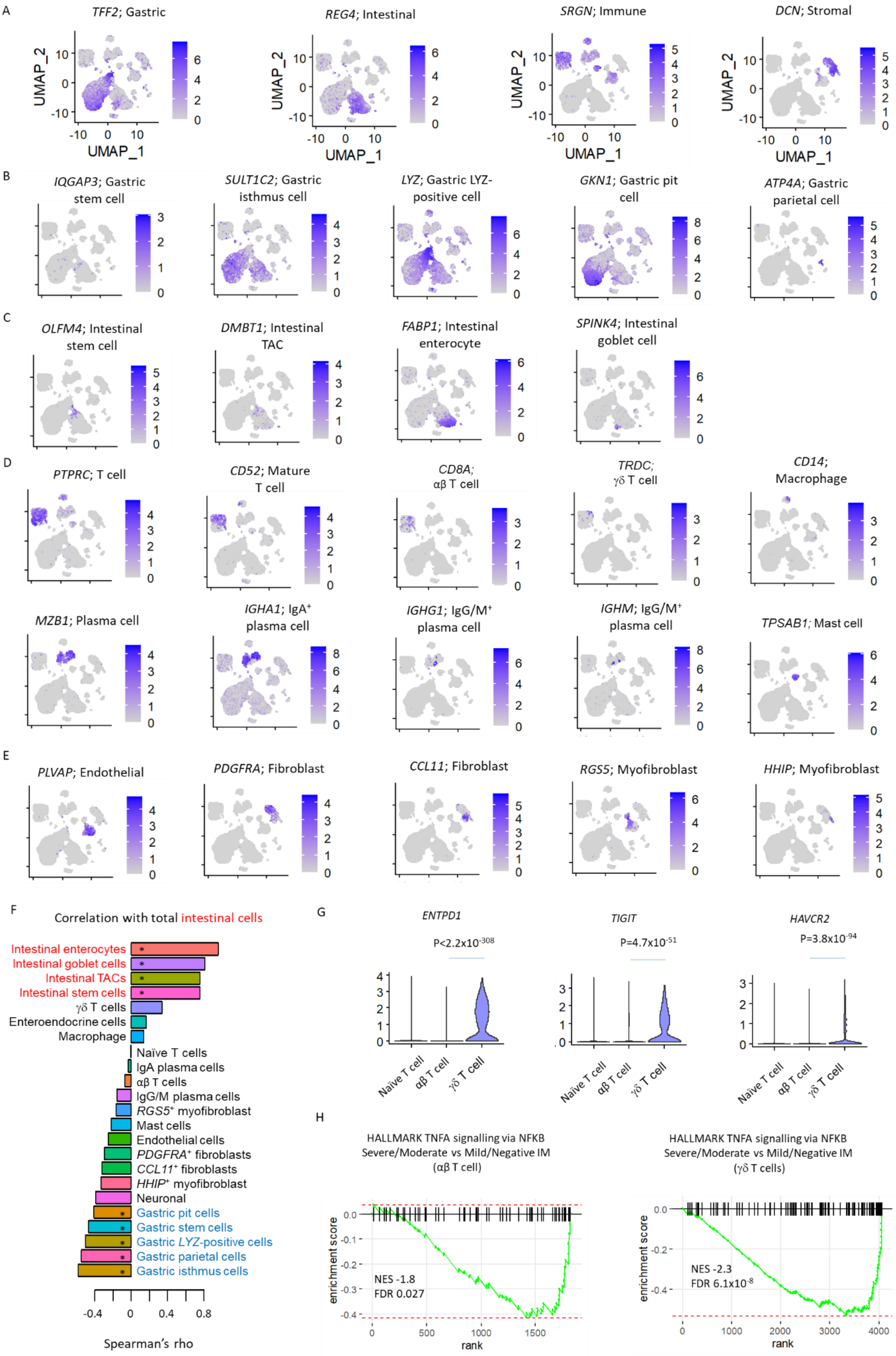
A
TFF2; Gastric
REG4; Intestinal
SRGN; Immune
DCN; Stromal
UMAP_1
UMAP_2
B
IQGAP3; Gastric stem cell
SULT1C2; Gastric isthmus cell
LYZ; Gastric LYZ-positive cell
GKN1; Gastric pit cell
ATP4A; Gastric parietal cell
C
OLFM4; Intestinal stem cell
DMBT1; Intestinal TAC
FABP1; Intestinal enterocyte
SPINK4; Intestinal goblet cell
D
PTPRC; T cell
CD52; Mature T cell
CD8A; αβ T cell
TRDC; γδ T cell
CD14; Macrophage
MZB1; Plasma cell
IGHA1; IgA+ plasma cell
IGHG1; IgG/M+ plasma cell
IGHM; IgG/M+ plasma cell
TPSAB1; Mast cell
E
PLVAP; Endothelial
PDGFRA; Fibroblast
CCL11; Fibroblast
RGS5; Myofibroblast
HHIP; Myofibroblast
F
Correlation with total intestinal cells
Intestinal enterocytes
Intestinal goblet cells
Intestinal TACs
Intestinal stem cells
γδ T cells
Enteroendocrine cells
Macrophage
Naïve T cells
IgA plasma cells
αβ T cells
IgG/M plasma cells
RGS5+ myofibroblast
Mast cells
Endothelial cells
PDGFRA+ fibroblasts
CCL11+ fibroblasts
HHIP+ myofibroblast
Neuronal
Gastric pit cells
Gastric stem cells
Gastric LYZ-positive cells
Gastric parietal cells
Gastric isthmus cells
Spearman's rho
G
ENTPD1
P<2.2x10-308
TIGIT
P=4.7x10-51
HAVCR2
P=3.8x10-94
Naïve T cell
αβ T cell
γδ T cell
H
HALLMARK TNFA signalling via NFKB
Severe/Moderate vs Mild/Negative IM
(αβ T cell)
NES -1.8
FDR 0.027
HALLMARK TNFA signalling via NFKB
Severe/Moderate vs Mild/Negative IM
(γδ T cells)
NES -2.3
FDR 6.1x10-8
enrichment score
rank

Figure S4. Annotation of single-cell RNAseq clusters. Related to Figure 3. (A) Marker genes for gastric (*TFF2*), intestinal (*REG4*), immune (*SRGN*) and stromal (*DCN*) lineage clusters. (B) Marker genes for gastric lineage cell clusters. (C) Marker genes for intestinal lineage cell clusters. (D) Marker genes for immune cell clusters. (E) Marker genes for stromal cell clusters (F) Correlation between cell type with intestinal cell-lineage proportions (intestinal stem cells, TACs, goblet cells and enterocytes). Intestinal cell types are in red text and gastric cell types are in blue text. Asterisk indicate significant Spearman correlation ($p<0.05$). (G) Comparison between immune cell exhaustion markers. (H) Down-regulation of the TNFα pathway in $\gamma\delta$ T cells of severe/moderate IM samples.

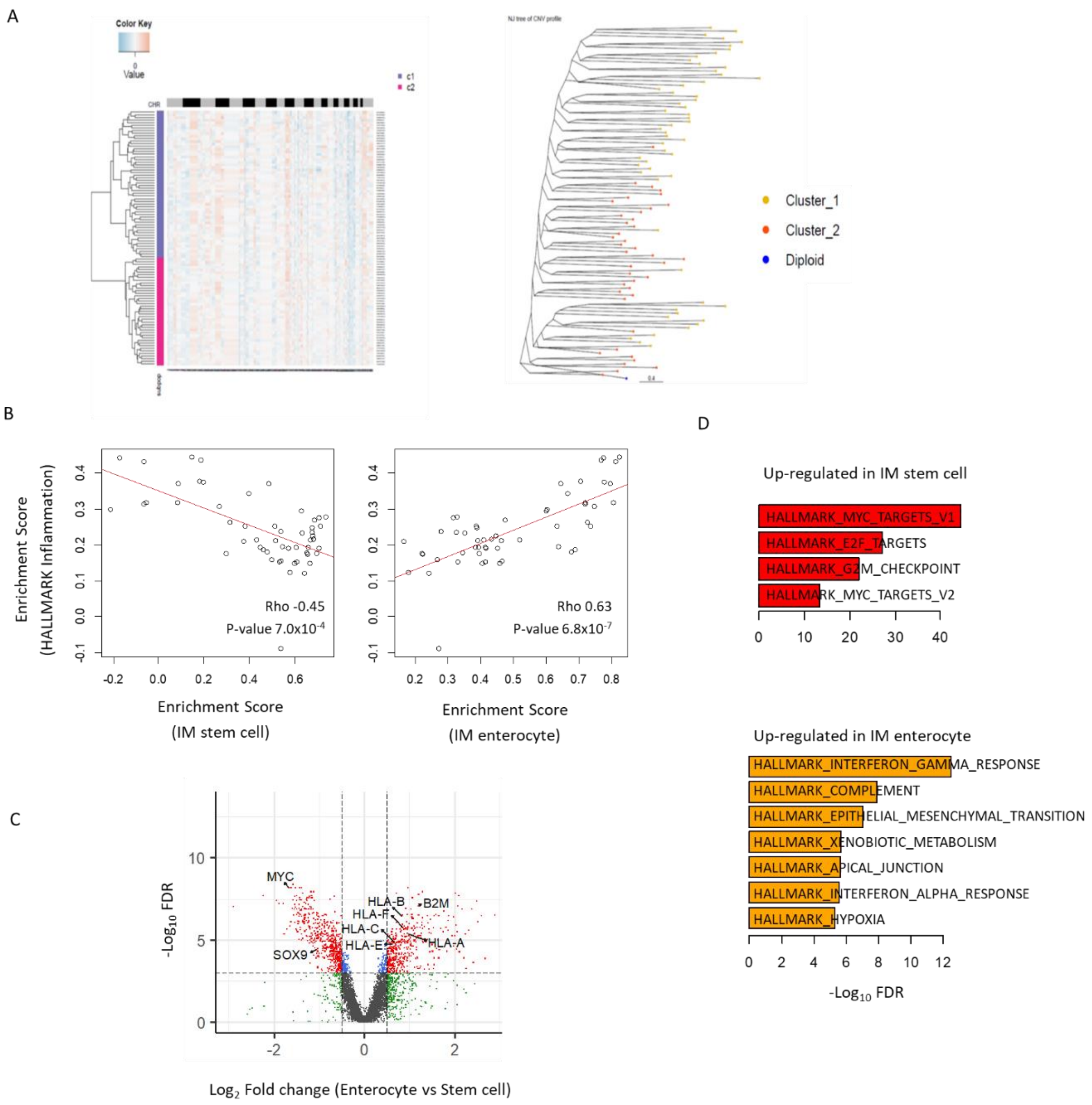

Figure S5. Comparison between IM stem cells, enterocytes and early GC. Related to Figure 4. (A) Copy number inference in GC epithelial single cells (left, a single GC case is depicted). Neighbour joining clustering of single cells using a diploid cell as root (right). Cell clusters with reduced copy number burden clustering closer to diploid cells were considered as early stage GC. (B) Spearman correlation between IM stem cell (left) and enterocyte (right) enrichment scores in DSP ROIs with HALLMARK inflammation enrichment scores. (C) Volcano plot showing significantly differentially expressed genes between enterocyte and stem cell regions. (D) Pathway analysis showing up-regulated gene expression in IM stem cells (upper) and enterocytes (lower).

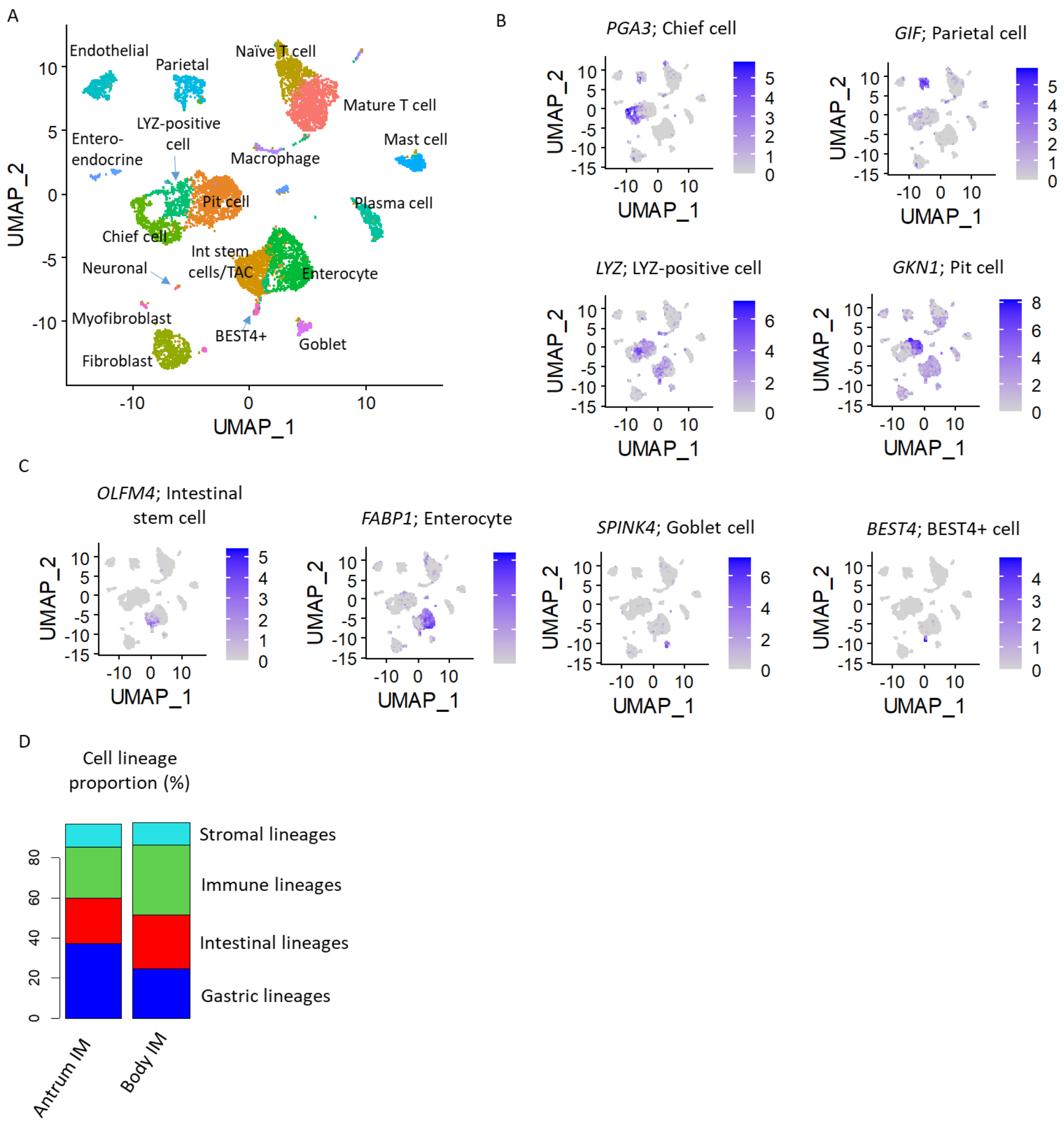

Figure S6. scRNAseq profiles of gastric body biopsies. Related to Figure 5. (A) Cell clusters identified from 6 gastric body samples (4 IMs and 2 normal; 10,176 cells) (left) and marker genes used to annotate (B) gastric and (C) intestinal cell types in antrum or body/cardia IMs. (D) Cell lineage proportions in antral and body IM.

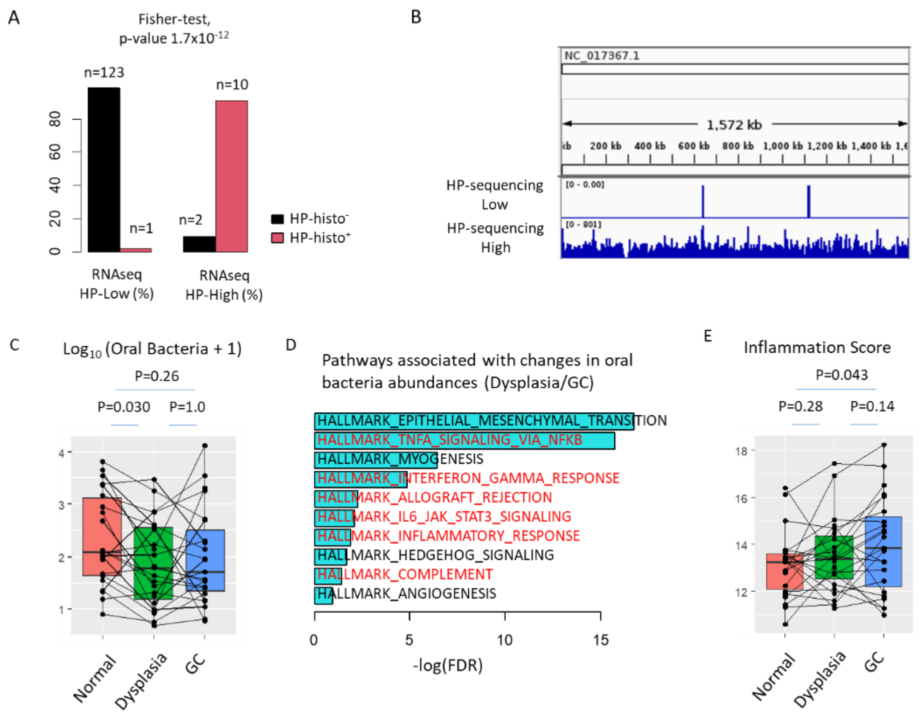

Figure S7. Microbial detection using Pathseq (RNAseq). Related to Figure 6. (A) Comparison between *Helicobacter*-high detection from RNAseq against Hp histological assessment. (B) IGV view of Hp genome (NC_017367.1) coverage from representative IM samples harbouring high levels (>1000 *Helicobacter* reads per 1 million reads mapping to human genome) and low levels of *Helicobacter* reads. (C) Abundance of oral bacteria in concurrent normal/dysplasia/GC samples. P-values were calculated using paired Wilcoxon tests. (D) Hallmark gene expression changes associated with changes in oral bacteria abundance (DESeq2), controlled by individual patients. (E) Inflammation scores in concurrent non-malignant/dysplasia/GC samples. P-values were calculated using paired Wilcoxon tests.

A

	p-value	Odds ratio	95% C.I.	
			Lower	Upper
All_Bacteria_Antrum	0.748	1.000	0.998	1.002
Oral_Bacteria_Antrum	0.465	0.380	0.028	5.104
Gastric_Bacteria_Antrum	0.529	0.095	0.000	144.137
Helicobacter_Antrum	0.749	1.000	0.998	1.002

B

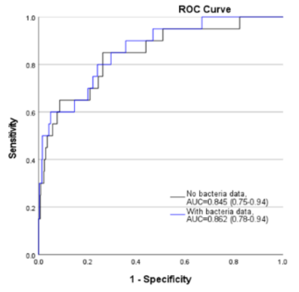

C

	Discovery Set			Validation Set		
	AUC	Sensitivity	Specificity	AUC	Sensitivity	Specificity
1	0.796	90%	68%	0.791	80%	78%
2	0.762	83%	68%	0.664	100%	100%
3	0.793	73%	87%	0.793	67%	86%
4	0.815	92%	78%	0.757	88%	83%
5	0.766	80%	85%	0.707	90%	80%
Average	0.787	84%	77%	0.742	85%	85%

D

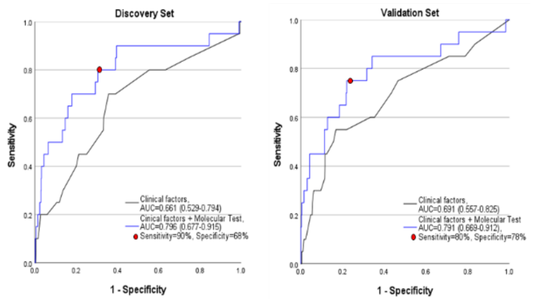

Figure S8. IM features associated with progression to dysplasia. Related to Figure 7. (A) Univariate logistical analysis using the abundance of all bacterial, oral bacterial, gastric bacterial and *Helicobacter* inferred from targeted DNA-seq. (B) ROC curves showing accuracy of prediction based on clinical factors and genomic features (mutation count, clone size, copy number variation) with (blue) or without (black) incorporation of the bacterial data. (C) Cross-validation analysis of the clinical-genomic model. (D) Representative cross-validation ROC curves used for internal verification. Area under the curves (AUCs) were used to determine detection power.
